# Algorithmic Management and Gig-Worker Sentiment over Time: A Comparative Analysis of Literature and Computational Evidence

**DOI:** 10.3390/bs16091709

**Published:** 2026-09-21

**Authors:** Nurettin Mert Batu, Hale Alan, Güray Tonguç, Neylan Kaya, Halil Özekicioğlu, Seda Sönmez, Hüseyin Topuz

**Affiliations:** 1 Department of Marketing, Faculty of Applied Sciences, Akdeniz University, Antalya 07070, Türkiye; mertbatu@akdeniz.edu.tr; 2 Department of Management Information Systems, Manavgat Faculty of Social Sciences and Humanities, Akdeniz University, Antalya 07600, Türkiye; 3 Department of Management Information Systems, Faculty of Applied Sciences, Akdeniz University, Antalya 07070, Türkiye; guraytonguc@akdeniz.edu.tr; 4 Department of Business Administration, Faculty of Economics and Administrative Sciences, Akdeniz University, Antalya 07070, Türkiye; 5 Department of International Trade and Logistics, Faculty of Applied Sciences, Akdeniz University, Antalya 07070, Türkiye; hozekicioglu@akdeniz.edu.tr; 6 Department of Finance and Banking, Faculty of Applied Sciences, Akdeniz University, Antalya 07070, Türkiye

**Keywords:** algorithmic management, algorithmic control, gig worker, gig economy, sentiment

## Abstract

Algorithmic management increasingly shapes how gig work is organised, evaluated, and experienced, yet its implications for workers’ affective responses remain fragmented across the literature. This study integrates a systematic review with computational analysis to examine how algorithmically mediated work is represented in research and expressed in online gig-worker discourse. The systematic review followed PRISMA guidelines and synthesised evidence on autonomy, fairness, transparency, evaluation, resource insecurity, and worker experience. The computational component analysed a corpus of 10,000 online texts collected over 12 consecutive months in 2025 from gig-worker-related digital communities and platforms. Sentiment and emotion were examined using NLP-based classification and term extraction, with temporal and contextual patterns assessed across the corpus. The computational analysis found that negative sentiment was more prevalent than neutral and positive sentiment, with frustration, anxiety, and anger among the most prominent expressed emotions. These patterns broadly corresponded with themes identified in the systematic review, particularly concerns regarding fairness, uncertainty, autonomy, transparency, and algorithmic control. However, the temporal findings represent changes in aggregate online discourse rather than within-person emotional trajectories or causal effects. The study concludes that computational analysis provides complementary evidence of how algorithmically mediated work is discussed and affectively expressed online, while individual lived experiences, psychological outcomes, and causal mechanisms require further investigation through longitudinal and mixed-method research.

## 1. Introduction

Algorithmic management has become an increasingly important topic as organizations make greater use of data and digital technologies to organize and manage work. Broadly defined, algorithmic management refers to the use of algorithms to perform managerial functions that were traditionally carried out by human supervisors, including task allocation, performance monitoring, worker evaluation, feedback provision, and the application of rewards or penalties (Lee et al., 2015; Jarrahi et al., 2021). These practices are particularly visible in the gig economy, where digital platforms connect workers with customers through short-term and flexible work arrangements (Shapiro, 2018). Despite the growing body of research on algorithmic management, existing studies have examined both its potential benefits, such as efficiency and standardization, and its potential drawbacks, including stress, uncertainty, and disengagement (Gong, 2025). Over the past decade, algorithmic management has become central to scholarly debates concerning autonomy, precariousness, and justice in the gig economy. Although platform-based work may provide workers with greater flexibility and access to income opportunities, it also changes how work is coordinated, evaluated, and controlled.

Algorithmic management within platform capitalism is also manifested through rankings, lists, classifications, ratings, and other digital traces generated through user interactions (Kornberger et al., 2017). This development can be situated within the broader tradition of Taylorism or scientific management. A fundamental objective of Taylorism was to systematically monitor workers, identify inefficiencies, and direct effort towards productivity. Digital Taylorism extends this principle using digital technologies to monitor, coordinate, and evaluate work and can be observed across a range of contemporary organizations (Park & Ryoo, 2023). In platform-based work, workers are increasingly coordinated and evaluated through algorithmic systems rather than solely through direct interaction with human supervisors. Unlike traditional Taylorist management, which relies primarily on hierarchical forms of control involving managers, workers, technologies, and organizational procedures, algorithmic management operates through a socio-technical network involving workers, digital platforms, algorithms, and data-driven protocols. Within this system, control can be exercised through feedback loops connecting workers, customers, and platform algorithms, enabling continuous monitoring, evaluation, and automated decision-making with limited direct managerial interaction (Stark & Pais, 2020; Kellogg et al., 2020).

Digital platforms increasingly use algorithms to determine which tasks workers receive, how their performance is evaluated, and, in some cases, whether they continue to have access to the platform (Rani & Furrer, 2021). These systems process large amounts of data and allow platforms to monitor workers continuously, often without direct interaction between workers and human managers (Jarrahi et al., 2021). Measures such as response times, task acceptance rates, customer ratings, and completion rates can affect workers’ access to jobs and income (Laeeque, 2025). As a result, the flexibility associated with gig work may exist alongside forms of control that are less visible than those found in traditional workplaces.

This combination of flexibility and control is one of the central issues in understanding platform work. Workers may decide when to work and, to some extent, which tasks to accept, but their activities are still shaped by platform rules and algorithmic systems. Previous research has consequently linked algorithmic management to issues such as autonomy, surveillance, information asymmetry, fairness, job security, and well-being (Rosenblat & Stark, 2016; Kellogg et al., 2020; Möhlmann et al., 2021; Vallas & Schor, 2020). The important difference is that control is increasingly embedded in the technology itself. Decisions that were once made through direct communication with a manager may now be made automatically, leaving workers with limited information about how or why particular decisions are reached.

The use of algorithmic management is not limited to a single type of platform or occupation. It has become common across areas such as food delivery, ride-sharing, online freelancing, accommodation services, and other forms of digitally mediated work (R. Liu & Yin, 2024; Keegan & Meijerink, 2025). The appeal of these platforms lies partly in their ability to coordinate large numbers of workers efficiently and respond quickly to changing demand. At the same time, this model places greater responsibility for organizing and monitoring work within digital systems. As algorithmic decision-making becomes more deeply integrated into everyday work, understanding how workers experience these systems has become increasingly important (Kellogg et al., 2020; Cameron et al., 2023).

The consequences of algorithmic management are not limited to working conditions or economic outcomes. The way algorithms allocate tasks, evaluate performance, determine rewards, or impose penalties can also shape how workers feel about their work. Experiences of unfair treatment, uncertainty, or constant monitoring may generate frustration, anxiety, or dissatisfaction, while greater predictability and perceived fairness may contribute to more positive responses. These emotional reactions provide an important indication of how workers interpret algorithmic control. This issue is particularly relevant for gig workers because they often have fewer opportunities to communicate their concerns through conventional organizational channels (Rosenblat & Stark, 2016; Vallas & Schor, 2020). Instead, workers frequently discuss their experiences in online communities, forums, and other digital spaces, creating a valuable source of naturally occurring textual data.

Although research on algorithmic management has expanded considerably, much of the literature has concentrated on questions of control, autonomy, surveillance, precariousness, justice, and organizational outcomes. There is comparatively less research on how workers emotionally respond to algorithmic decisions and, more importantly, how these responses may change over time. This temporal dimension deserves greater attention because workers’ perceptions are not necessarily fixed. Changes in platform policies, pricing systems, performance requirements, workloads, or account-management practices may influence how workers feel about algorithmic management at different points in time. Looking at these changes longitudinally can therefore provide a more complete picture of the relationship between algorithmic management and worker experience.

Sentiment analysis provides a useful way of examining this dimension. As a natural language processing technique, sentiment analysis is used to identify and classify the emotional orientation expressed in textual data, including online reviews, social media posts, discussion forums, and user comments (B. Liu, 2012; Medhat et al., 2014; Zhang et al., 2018). It can distinguish between broad categories such as positive, neutral, and negative sentiment and can also be used to identify more specific emotional states (Cambria et al., 2017; Balcıoglu & Artar, 2024). When applied to gig-worker texts, sentiment analysis makes it possible to examine not only what workers say about algorithmic management but also the emotional tone associated with those experiences.

Against this background, the present study brings together two perspectives that are often examined separately. First, it reviews the existing literature to identify the main concepts and themes associated with algorithmic management and gig-worker sentiment. Second, it examines workers’ own textual expressions using sentiment analysis. Bringing these two sources of evidence together makes it possible to assess whether the concepts emphasized in the literature are also reflected in workers’ real-world experiences. The study also considers how worker sentiment develops over time, rather than treating emotional responses as static.

The study therefore focuses on the relationship between algorithmic management and the lived experiences of gig workers. More specifically, it examines whether the concerns identified in the academic literature—such as autonomy, control, fairness, uncertainty, and well-being—are reflected in workers’ expressed sentiments and whether the intensity or direction of these sentiments changes over time. By comparing literature-based concepts with workers’ actual textual expressions, the study aims to provide a more grounded understanding of how algorithmic management is experienced in everyday platform work. To address this gap, the present study develops a priori framework in which algorithmic management is theorized to influence workers’ justice appraisals, perceived resources, and causal attributions, which in turn shape expressed affective states and behavioral responses. The framework further considers worker- and platform-level conditions that may moderate these relationships. This theoretical architecture guides systematic literature review and the subsequent computational sentiment analysis.

Accordingly, the study addresses the following research questions:

RQ1: How are gig workers’ expressed sentiments and affective responses associated with algorithmic management and control over time?

RQ2: To what extent do the concepts identified in the literature on gig worker sentiment correspond to sentiments and affective responses expressed in gig-worker online narratives?

RQ3: What similarities and discrepancies emerge between theoretically derived constructs and empirically observed sentiments and affective responses in gig-worker narratives?

RQ4: How can the integration of literature-based concepts and computationally observed sentiments contribute to a more comprehensive conceptual framework for understanding gig workers’ experiences of algorithmic management?

## 2. Theoretical Framework and a Priori Model

Algorithmic management should not be conceptualized as inherently beneficial or detrimental to gig workers. While a substantial body of literature emphasizes surveillance, opacity, control, and reduced autonomy (Rosenblat & Stark, 2016; Wood et al., 2019), algorithmic systems may also generate coordination efficiencies, improve information flows, enhance flexibility, and expand access to work opportunities when designed and implemented in transparent ways (Kellogg et al., 2020; Lee et al., 2015; Möhlmann & Zalmanson, 2017). For instance, automated task allocation can reduce search and matching costs, real-time information systems can support decision-making regarding tasks and earnings, and data-driven scheduling mechanisms may increase temporal flexibility by enabling workers to choose when and where to work (Lee et al., 2015; Sundararajan, 2016). Similarly, transparent performance feedback and clearly defined reward structures may enhance workers’ understanding of platform expectations and improve their capacity for planning and coordination.

Accordingly, this study conceptualizes the effects of algorithmic management as contingent rather than inherently positive or negative. Identical technological infrastructures may function as enabling mechanisms when they enhance predictability, informational clarity, coordination, and perceived autonomy, yet may simultaneously operate as constraining mechanisms when they intensify surveillance, increase uncertainty, or expose workers to opaque and unilateral sanctions. This duality constitutes the conceptual foundation of the a priori theoretical model developed in this study.

The proposed framework integrates insights from algorithmic management research with organizational justice theory, Conservation of Resources (COR) Theory, Self-Determination Theory (SDT), emotional labor scholarship, and the complementary notion of affective governance. These theoretical perspectives are not treated as competing or post hoc explanatory devices; rather, they are positioned as analytically distinct but interrelated layers within a unified conceptual model:

Algorithmic-management features → cognitive/evaluative mechanisms → emotion-related textual expressions → potential behavioural responses.

These relationships are further conditioned by contextual factors, including occupational category, platform type, income dependence, worker experience, institutional environment, and access to human appeal or dispute-resolution mechanisms.

### 2.1. Algorithmic Management Features

The first level of the model concerns the structural and functional characteristics of algorithmic management systems. These include algorithmic opacity, automated evaluation systems, dynamic pricing mechanisms, surveillance and performance monitoring infrastructures, task allocation and matching systems, ranking and reputation systems, and automated enforcement mechanisms such as deactivation or account restriction (Kellogg et al., 2020; Lee et al., 2015; Rosenblat & Stark, 2016; Möhlmann et al., 2021).

Importantly, algorithmic management is not treated as a monolithic construct. Different system components perform distinct functions with potentially divergent implications for worker experience. For example, matching and scheduling systems may primarily enhance coordination and reduce uncertainty, whereas automated evaluation, ranking, and punitive enforcement systems may exert stronger forms of behavioural control. Consequently, the theoretical implications of algorithmic management depend on the specific configuration, transparency, and implementation of these system components.

### 2.2. Cognitive and Evaluative Mechanisms

The second level of the model captures the cognitive and evaluative processes through which algorithmically mediated working conditions are interpreted.

Organizational justice theory provides a framework for understanding perceptions of distributive and procedural fairness, including the extent to which outcomes, workloads, evaluations, and decision-making processes are perceived as equitable, consistent, and explainable (Greenberg, 1987; Colquitt et al., 2001). In platform work contexts, algorithmic opacity, limited explanation of decisions, and restricted access to appeal mechanisms are particularly salient in shaping procedural justice perceptions (Rosenblat & Stark, 2016; Möhlmann & Zalmanson, 2017).

Conservation of Resources (COR) Theory conceptualizes worker responses in terms of perceived gains or losses in valued resources, including income stability, time, autonomy, and continued access to platform-mediated work (Hobfoll, 1989, 2001; Hobfoll et al., 2018). Within this framework, unpredictable earnings, sudden deactivation, fluctuating demand, or unstable task allocation are interpreted as potential resource loss or threat, which may trigger defensive or compensatory responses.

Self-Determination Theory (SDT) further specifies the role of basic psychological needs—autonomy, competence, and relatedness—in shaping motivational and evaluative responses (Deci & Ryan, 2000; Ryan & Deci, 2017). Algorithmic systems may support autonomy by increasing temporal and spatial flexibility, yet simultaneously constrain it when workers experience limited discretion over task selection or scheduling. Similarly, transparent feedback mechanisms may enhance perceived competence, whereas opaque or inconsistent evaluation systems may undermine it.

Taken together, these theoretical perspectives capture distinct but complementary evaluative dimensions—fairness, resource security, and psychological need satisfaction—through which algorithmic management is interpreted.

### 2.3. Emotion-Related Textual Expressions

The third level of the model concerns the linguistic articulation of affect in digital discourse. The computational analysis focuses on sentiment polarity and emotion-related textual expressions, including but not limited to frustration, anger, anxiety, satisfaction, hope, and resignation.

These indicators are explicitly conceptualized as textual and discursive manifestations of affect rather than direct measures of internal emotional states. Sentiment analysis and emotion classification techniques capture systematic patterns in language use but do not provide valid inferences about psychological well-being, clinical conditions, or the intensity of lived emotional experience (Pang & Lee, 2008; B. Liu, 2012; Hovy & Spruit, 2016; Mohammad, 2016). Moreover, online expressions are shaped by multiple communicative and contextual factors, including rhetorical strategies, humour, sarcasm, community norms, and platform-specific conventions.

Accordingly, the empirical focus is on how affect is expressed, structured, and distributed in online discourse, rather than on measuring individual-level emotional states.

### 2.4. Emotional Labor and Affective Governance

In this study, affective governance is not proposed as a replacement for algorithmic control, organizational justice, emotional labor, or related perspectives. Instead, it refers to the way algorithmically mediated work arrangements can structure the conditions under which affective reactions become salient, communicated, and repeatedly negotiated in workers’ online discourse (Kellogg et al., 2020; Rosenblat & Stark, 2016).

The concept adds a specific communicative dimension to established theories of algorithmic control. Algorithmic control explains how platforms use data-driven systems to allocate tasks, evaluate performance, monitor behaviour, and impose consequences (Kellogg et al., 2020; Lee et al., 2015). Organizational justice explains how workers evaluate the fairness of these arrangements across distributive, procedural, and interactional dimensions (Colquitt, 2001; Greenberg, 1987). Conservation of Resources (COR) Theory highlights perceived threats to valued resources such as income stability, time, and employment continuity (Hobfoll, 1989), while Self-Determination Theory emphasizes autonomy, competence, and relatedness as fundamental psychological needs that may be constrained in algorithmically mediated work (Deci & Ryan, 2000). Emotional labor theory further highlights the management and expression of emotion as part of work performance in service and platform contexts (Hochschild, 1983; Grandey, 2000). Affective governance focuses on what happens at the intersection of these processes: how recurring algorithmic uncertainty, evaluation, reward, sanction, and limited procedural recourse can make particular affective concerns more visible and recurrent in workers’ public accounts of platform work.

This definition places clear boundaries around the concept. Affective governance does not imply that platforms directly control workers’ internal emotions, nor does it imply that negative online sentiment is evidence of psychological distress. Its observable implication in the present study is more limited: algorithmically mediated events that involve uncertainty, reward, evaluation, fairness, or sanction may be associated with recurring emotion-related language and evaluative sentiment in online discussions (Rosenblat & Stark, 2016; Wood et al., 2019). Whether such discursive patterns correspond to changes in individual emotional states requires direct worker-level evidence and is therefore beyond the scope of the present study.

Affective governance is thus treated as a complementary interpretive concept that connects the structural operation of algorithmic control with the affective and communicative dimensions of platform work. Its distinctive contribution lies in directing attention to how algorithmically produced conditions become objects of affective interpretation and public expression, rather than claiming that algorithms directly determine workers’ emotional states.

Emotional labor theory provides a complementary lens for understanding how emotions are managed, displayed, and regulated in service-oriented and platform-mediated work environments (Hochschild, 1983; Grandey, 2000). In gig work contexts, algorithmic management and pervasive digital monitoring shape the conditions under which workers perform, are evaluated, and interact with platform-mediated systems. These mechanisms can influence workers’ autonomy, working conditions, and capacity to respond collectively to platform governance (Tassinari & Maccarrone, 2020; van Doorn, 2020).

The concept of affective governance is introduced as an additional analytical layer that captures how algorithmically mediated working conditions become recurrent objects of affective interpretation and public articulation in digital spaces. Importantly, affective governance does not imply direct manipulation or control of workers’ internal emotional states. Instead, it refers to the structural conditions under which algorithmic systems—through uncertainty, visibility, evaluation, and sanctioning mechanisms—generate recurring affect-laden discourses.

This study’s main analytical contribution is that it connects algorithmic control systems with the ways people emotionally communicate and respond to them. While algorithmic management structures organize and regulate work processes, evaluative perspectives help us understand how workers make sense of these systems in practice. At the same time, research on algorithmic power and platform governance shows that these interpretations and reactions are not formed in isolation; instead, they are shaped, shared, and reinforced through everyday interactions within digital environments (Bucher, 2018; Gillespie, 2018).

In this study, the observable implication of affective governance is limited to the salience, recurrence, and distribution of emotion-related textual expressions, rather than any claim regarding psychological regulation or emotional causation.

### 2.5. Potential Behavioural Responses

The framework also considers potential behavioral responses associated with workers’ evaluations and affective responses to algorithmic management. These may include compliance, voice, resistance, increased work effort, strategic adaptation, platform switching, and, in some circumstances, withdrawal or exit (Wood et al., 2019; Vallas & Schor, 2020). Such responses should be understood as potential forms of worker agency rather than as outcomes directly established by the present study. However, these behavioural outcomes are treated as theoretical extensions rather than empirically observed variables. Given that the computational dataset primarily captures textual discourse rather than longitudinal behavioural trajectories, these responses are not directly tested but are instead included as analytically plausible implications of the proposed model.

### 2.6. Contextual Conditions

The relationships proposed in the framework may vary across contextual and worker-level conditions, including occupational category, platform type, workers’ economic dependence on platform income, prior experience with platform work, institutional and regulatory environments, and the availability of human-mediated mechanisms for contesting or appealing algorithmic decisions (Wood et al., 2019; Veen et al., 2020; Newlands, 2021). These conditions may shape how workers perceive algorithmic control, evaluate its fairness and transparency, and respond to platform-mediated decisions. For instance, algorithmic transparency may be experienced as enabling under conditions of low income dependence and high occupational flexibility, whereas the same transparency may be perceived as constraining under conditions of high dependency and limited alternative employment opportunities. Similarly, accumulated platform experience may facilitate adaptation to algorithmic systems, while prolonged exposure to perceived unfairness or instability may intensify negative evaluative and affective responses. These moderating effects are conceptualized as contingent rather than deterministic.

## 3. Methodology

This study adopts a mixed-methods research design that integrates qualitative and quantitative approaches to provide a comprehensive understanding of the emotional consequences of algorithmic management in gig work. Specifically, the study employs a sequential two-stage design comprising (1) a PRISMA-based systematic literature review and (2) a computational sentiment analysis using Natural Language Processing (NLP).

The first stage consists of a systematic literature review conducted in accordance with the PRISMA 2020 guidelines. Systematic reviews play a critical role in consolidating evidence, identifying research priorities, evaluating theoretical developments, and revealing limitations in existing studies that warrant further investigation (Page et al., 2021). The review synthesises existing research on algorithmic management, gig work, platform governance, and emotions in digital labour. Particular attention is given to the application domains of sentiment analysis, model performance evaluation approaches, analytical techniques, evaluation metrics, and programming environments. In addition to summarising the current state of knowledge, the review identifies methodological gaps, emerging challenges, and promising directions for future research.

The second stage applies Natural Language Processing (NLP) techniques to examine gig workers’ perceptions of algorithmic management using a dataset of 10,000 worker-generated texts collected over a 12-month period from Reddit communities in which gig workers discussed their experiences with platforms such as Grab, Bolt, Gojek, DoorDash, Yemeksepeti, Uber, and Getir. Sentiment analysis was used to classify workers’ narratives according to their sentiment polarity (positive, negative, and neutral), while emotion analysis was conducted to identify the emotional patterns expressed in relation to algorithmic management practices. As a subfield of NLP, sentiment analysis enables the extraction of opinions, emotions, attitudes, and behavioural intentions from unstructured textual data (Chen et al., 2018). To complement the computational findings, thematic analysis was conducted to identify recurring themes related to algorithmic control mechanisms and workers’ lived experiences. By integrating evidence from the systematic literature review with computational sentiment and emotion analysis and qualitative thematic interpretation, the study provides a more comprehensive understanding of how gig workers express and interpret affective responses to algorithmic management. This mixed-methods approach enables both the synthesis of existing knowledge and the empirical examination of large-scale textual data, thereby offering robust theoretical and practical insights into digitally mediated work environments.

### 3.1. Data Collection for PRISMA-Based Systematic Analysis

Studies were included if they met all of the following criteria: (1) focused on gig work, platform work, or app-based labour; (2) examined algorithmic management and algorithmic control; (3) addressed workers’ emotions, perceptions, experiences, or affective responses; (4) were published between January 2010 and December 2025; and (5) were published in English-language peer-reviewed academic journals or conference proceedings. Search terms included combinations of: “algorithmic management”, “algorithmic control”, “gig worker”, “platform labor”, “digital labor”, “sentiment”, “emotion”, “worker experience”, “platform governance”. The literature search was conducted across seven academic databases: Scopus, Web of Science, PubMed, Google Scholar, the Social Science Research Network (SSRN), ACM Digital Library, and IEEE Xplore. These databases were selected to provide broad coverage of research on gig work, platform labour, algorithmic management, and worker experiences across management, social science, health, and computer science disciplines. The number of records retrieved from each database is reported in Table 1.

**Figure 1:**
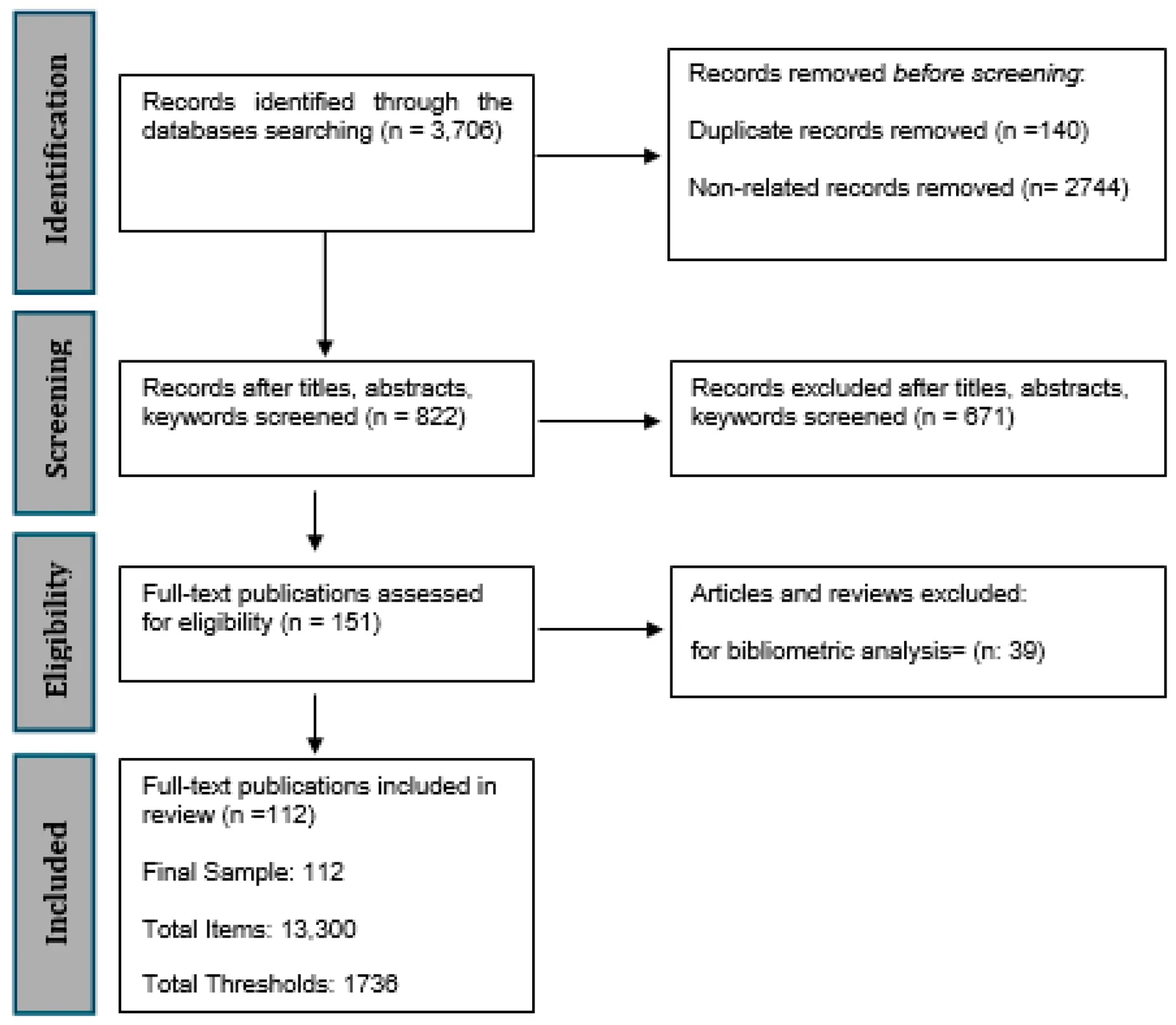
Preferred Reporting Items for Systematic Reviews and Meta-Analyses (PRISMA) flow diagram.

As shown in Table 1, the largest number of records was retrieved from the IEEE Xplore database (634), followed by PubMed (619), Scopus (602), Google Scholar (598), Web of Science (582), SSRN (559) and ACM DL (112). Figure 1 presents a PRISMA (Preferred Reporting Items for Systematic Reviews and Meta-Analyses) flow diagram summarizing the selection process. The figure details the four main phases—identification, screening, eligibility, and inclusion.

The PRISMA flow diagram (Figure 1) summarises the identification, screening, eligibility, and inclusion stages of the review. A total of 3706 records were identified across the selected databases. Following the full-text assessment, 151 records were excluded because they did not satisfy one or more of the inclusion criteria, while 112 studies met all eligibility requirements and were included in the final systematic review corpus. The numbers reported in the PRISMA diagram, Methods, Results, and Supplementary Materials were cross-checked to ensure consistency across all sections of the manuscript.

### 3.2. Data Collection and Construction of the Computational Corpus

The computational component of the study was based on publicly accessible Reddit content concerning gig and platform work during the period 1 January–31 December 2025. The data-generation process consisted of three distinct stages: (1) retrieval of potentially relevant records, (2) eligibility screening and data cleaning, and (3) construction of the final analytical corpus.

During the twelve-month observation period, 24,258 potentially relevant records were initially retrieved from seven predefined platform-specific Reddit communities: r/BoltDrivers, r/doordash_drivers, r/GetirKuryeleri, r/GojekDrivers, r/GrabDrivers, r/uberdrivers, and r/kurye. These 24,258 records constituted the raw retrieval pool and were not treated as the analytical sample.

The raw retrieval pool was subsequently screened using predefined inclusion and exclusion criteria. A record was retained when the textual content provided sufficient evidence that the author was discussing their own experience as a gig or platform worker. Evidence included first-person descriptions of performing platform-mediated work, accepting or rejecting tasks, discussing earnings or incentives, describing ratings or performance evaluations, discussing algorithmic task allocation or routing, or reporting automated warnings, suspension, or deactivation.

Records were excluded when they consisted primarily of customer-authored content, promotional or advertising material, news reports, general discussions without evidence of worker experience, duplicate or reposted material, or content for which gig-worker status could not be established with sufficient confidence. Automated or clearly non-user-generated material was also excluded where identifiable.

Following the screening and eligibility assessment, 10,000 records met all predefined eligibility criteria, corresponding to 41.25% of the raw retrieval pool. Thus, 14,258 records (58.75%) were excluded during the screening and eligibility process. Importantly, all 10,000 eligible records were retained in the final analytical dataset. Consequently, the final analytical corpus was not created by selecting an unexplained target number from the eligible records; rather, the final corpus comprises the complete set of records that satisfied the predefined eligibility criteria.

According to Table 2, the raw retrieval pool consisted of 24,258 potentially relevant Reddit records collected during January–December 2025. Following duplicate removal, relevance assessment, worker-status verification, and exclusion of customer-authored, promotional, news, and other ineligible material, 10,000 records met the predefined eligibility criteria. All eligible records were retained; therefore, no additional subsampling was conducted after eligibility screening.

Platform/community was retained as an important sampling and analytical dimension. The final dataset contains approximately balanced representation across the seven predefined platform communities. The exact distribution was: Bolt, 1368 posts (13.68%); DoorDash, 1487 (14.87%); Getir, 1458 (14.58%); Gojek, 1382 (13.82%); Grab, 1403 (14.03%); Uber, 1393 (13.93%); and Yemeksepeti, 1509 (15.09%). These proportions should not be interpreted as estimates of the natural prevalence of Reddit activity across platforms. Rather, they reflect the study’s deliberate effort to maintain adequate representation of the seven platform contexts in the analytical corpus.

According to Table 3, the monthly composition of the corpus was also retained. Monthly platform composition was examined separately to determine whether changes in sentiment could be explained by shifts in the relative contribution of different platform communities. The distribution was structured to preserve variation across both platforms and months. A total of 24,258 raw records were initially retrieved, after which records that did not meet the predefined eligibility criteria, including duplicates, irrelevant content, non-worker posts, insufficient textual content, and records outside the observation period, were excluded. The remaining 10,000 eligible posts constitute the final analytical dataset. As shown in the table, platform-level frequencies range from 1368 posts for Bolt to 1509 posts for Yemeksepeti, while monthly observations range from 767 posts in February to 881 posts in January. The final row and column confirm that the platform-level and monthly distributions both sum to exactly 10,000 observations. Thus, the table provides a transparent account of how the final analytical corpus was distributed across platforms and time and clarifies that 10,000 refers to the final eligible sample rather than the initial number of retrieved records.

While pseudonymous user identifiers were retained across the 12-month observation period (January–December 2025, with 4908 unique pseudonymous authors contributing 10,000 posts over the observation period), the dataset constitutes repeated observational cross-sections of online discourse rather than a prospective, balanced individual panel. Precise platform entry dates, individual job tenure, and continuous within-person psychological development cannot be definitively established. Consequently, observed shifts in sector sentiment reflect aggregate temporal patterns in discussion spaces rather than verified within-person emotional trajectories. The final analytical dataset comprised 10,000 observations generated by 4908 unique pseudonymous authors. The mean number of observations per author was 2.04, with a median of 2 and a maximum of 8 observations from a single author. The distribution of contributions indicates that 1914 authors contributed one observation, 1604 contributed two, 874 contributed three, 369 contributed four, 112 contributed five, 28 contributed six, 4 contributed seven, and 3 contributed eight observations. Thus, the dataset should not be interpreted as representing 10,000 independent workers. Rather, the post constitutes the unit of analysis, while the author represents a repeated-observation clustering level. Pseudonymous author identifiers were retained solely to identify repeated contributions and were not used to reconstruct individual identities. Accordingly, within-author dependence was considered in the statistical analysis, and the results are interpreted as patterns of repeated online discourse rather than as independent observations from 10,000 individual gig workers.

Importantly, the final corpus of 10,000 posts should not be interpreted as a simple convenience sample or as evidence that the seven platforms represent equivalent user populations. Platform-specific differences in worker composition, geographic coverage, moderation practices, and posting behaviour may affect the nature and distribution of the observed textual data. These differences were therefore treated as relevant characteristics of the data rather than assumed to be negligible. Accordingly, platform and month were retained as distinct analytical dimensions rather than being collapsed into a single undifferentiated source category. This approach enables the analysis to account for cross-platform heterogeneity and temporal variation while avoiding the assumption that observed sentiment patterns are directly comparable across platforms without contextual qualification. The resulting findings should therefore be interpreted as patterns in online gig-worker discourse across heterogeneous platform environments rather than as estimates that are necessarily representative of the broader gig-worker population.

A hybrid computational approach was employed by combining transformer-based and lexicon-based sentiment analysis techniques. Specifically, a transformer-based classifier fine-tuned from the BERT architecture was used to classify textual data into positive, neutral, and negative sentiment categories. To complement this classification, the lexicon-based Valence Aware Dictionary and Sentiment Reasoner (VADER) was employed to quantify sentiment intensity. Subsequently, linguistic analysis was conducted to identify discrete emotional categories, including anger, frustration, relief, joy, and anxiety. Integrating these complementary techniques improves the accuracy and robustness of sentiment detection, particularly when analyzing informal, user-generated online text. We linked the 12-month study period to key platform events such as payment changes, announcements, and algorithm updates. However, we do not treat timing alone as evidence of causality. Posts were labeled as event-related only if they explicitly mentioned the event using clear keywords or contextual references. Posts from the same period without such references were included in the overall analysis but not considered direct reactions to the event. We then compared sentiment between event-related and non-event-related posts and examined changes before and after each event when data allowed. These analyses are used to identify patterns over time, not causal effects.

Overall, we interpret results cautiously. Increases in negative sentiment after an event are described as temporal associations unless users clearly discuss the event itself. Even then, other factors may also explain the observed changes.

#### 3.2.1. Longitudinal Analysis and Temporal Modelling

We tracked gig-worker sentiment over 12 months using 10,000 texts from 7 target platform communities on Reddit (Grab, Bolt, Gojek, DoorDash, Yemeksepeti, Uber, Getir). Each text was assigned to its posting month, and we checked monthly distributions to ensure results weren’t driven by uneven sample sizes. Beyond descriptive analysis, we applied an interrupted time-series (ITS) model to capture changes in sentiment over time. Negative sentiment was the main outcome since it was the most common, while positive and neutral sentiment were also analysed. Time was coded from 1 to 12 to capture overall trends and potential shifts linked to key events.

When relevant platform or external events were identified, they were treated as interruption points. The model estimated both immediate changes in sentiment and changes in trend after these events:

Y_t_ = β_0_ + β_1_(Time_t) + β_2_(Intervention_t) + β_3_(Time After Intervention_t) + ε_t_

Here, β_0_ is the baseline level, β_1_ the pre-event trend, β_2_ the immediate shift, and β_3_ the post-event trend change.

Since monthly sample sizes varied, we used weights where needed and adjusted standard errors for temporal dependence. We also reported 95% confidence intervals and checked model assumptions using autocorrelation and residual diagnostics.

To ensure robustness, we repeated analyses using different sentiment outcomes, excluded months with extreme sample sizes, tested alternative trend specifications, and compared results with simple descriptive trends. Overall, these checks confirmed that the findings reflect real temporal patterns rather than random fluctuations or sampling imbalance. 

**Figure 2:**
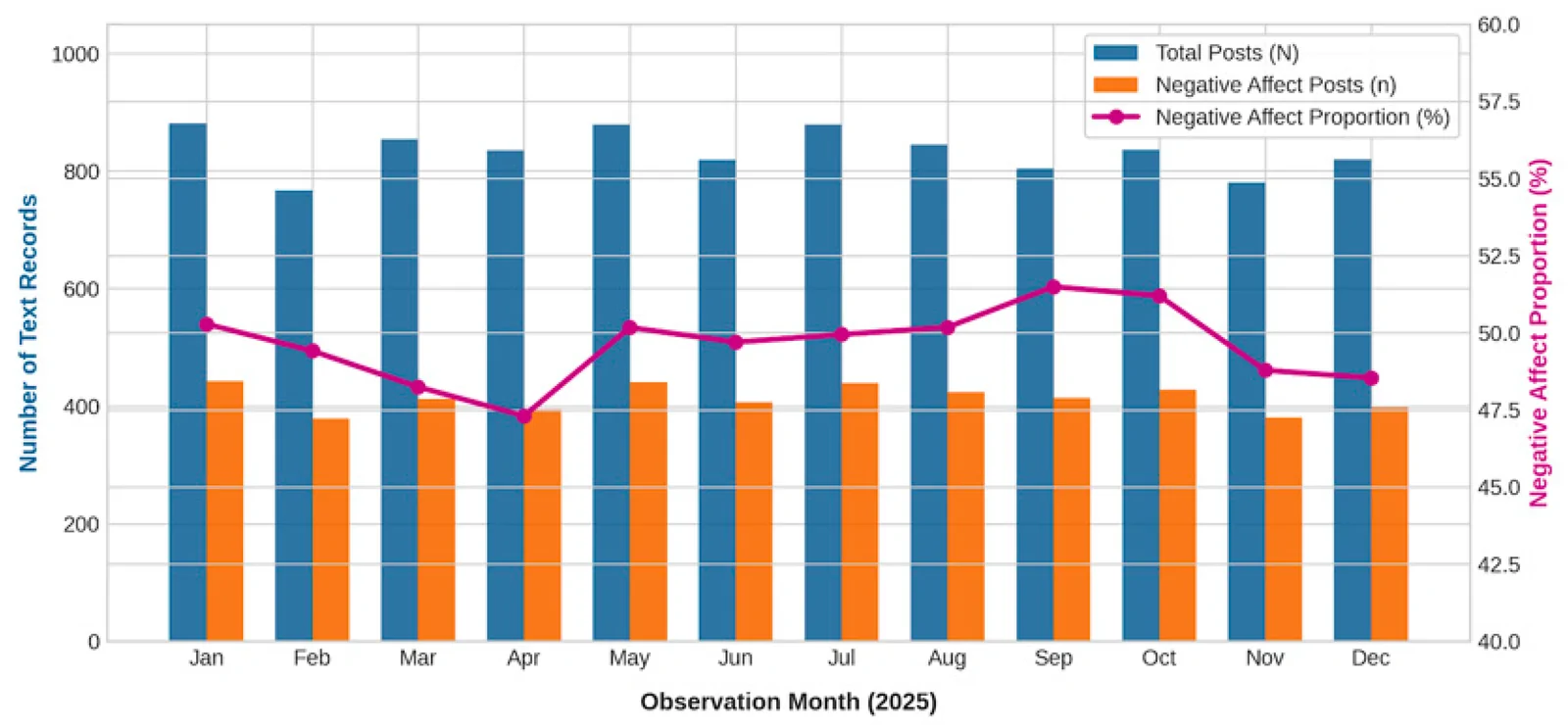
Monthly Volume of Gig Worker Text Data & Negative Affect Proportion (Jan–Dec 2025).

Table 4 presents the monthly empirical distribution of the corpus, along with descriptive statistics and visualizations of gig workers’ text volume and expressed negative affect over the 12-month observation period from January to December 2025. These analyses capture temporal variation in aggregate online discourse (Figure 2). They should not, however, be interpreted as evidence of within-worker emotional change or individual-level longitudinal trajectories. 

#### 3.2.2. Change-Point Detection & Multilevel Modeling (MLM)

Nonparametric CUSUM Change-Point Detection: We ran a Cumulative Sum (CUSUM) test across the 365 daily sentiment means to discover structural breaks without prior assumption. The peak deviation occurred on 12 April 2025. A Welch’s *t*-test comparing pre-break ($\bar{Y}=+0.0130$) vs. post-break ($\bar{Y}=+0.0013$) daily distributions showed no statistically significant mean shift (*t* = 0.8826, *p* = 0.3787), confirming trend structural stability.

Multilevel Mixed-Effects Modeling: To account for hierarchical grouping, we specified a two-level linear mixed model with random intercepts by platform (*k* = 7). The resulting Intraclass Correlation Coefficient (ICC < 0.001) showed that platform-level variance accounts for less than 0.1% of total sentiment dispersion, confirming that worker sentiment is driven primarily by individual-level post experiences rather than platform clustering.

#### 3.2.3. Exploratory Temporal Association Analysis: Interrupted Time-Series (ITS) Model

To examine whether changes in aggregate sentiment coincided temporally with externally identified platform events, we conducted an exploratory Interrupted Time-Series (ITS) analysis. The purpose of this analysis was to assess temporal associations and potential changes in level or trend around predefined event dates, rather than to establish causal effects or eliminate selection bias and endogeneity. Daily aggregated sentiment scores were regressed on pre-intervention time trends, intervention indicators for externally verified events, post-intervention time counters, and daily post volumes as a control covariate. Heteroskedasticity and autocorrelation (HAC)-robust standard errors were calculated using Newey–West estimators with a 7-day maximum lag.

The ITS specification was used as a sensitivity and robustness check of the descriptive temporal patterns. The model estimated the baseline trend, the immediate change associated temporally with an event, and the subsequent change in trend:

*Y_t_* = *β*_0_ + *β*_1_(*Time_t*) + *β*_2_(*Intervention_t*) + *β*_3_(*Time After Intervention_t*) + *ε_t_*

where β_0_ represents the baseline level, β_1_ the pre-event temporal trend, β_2_ the estimated change in level at the event point, and β_3_ the change in trend following the event.

The ITS estimates did not indicate statistically significant immediate changes or subsequent slope changes around the examined platform events. These null findings provide no statistical evidence of abrupt event-associated shifts in aggregate sentiment. They should not, however, be interpreted as evidence that platform events had no effect on individual workers, because the analysis is based on aggregate online discourse and does not provide individual-level causal identification. Accordingly, the ITS results are interpreted as exploratory evidence concerning temporal association and aggregate discourse stability.

#### 3.2.4. Sampling, Representativeness, and Source Heterogeneity

The corpus consists of 10,000 posts collected from 7 target platform communities on Reddit (Grab, Bolt, Gojek, DoorDash, Yemeksepeti, Uber, Getir) and should be interpreted as online discourse on platform work rather than a representative sample of gig workers. These sources differ in user populations, moderation rules, and communication norms, which may shape how experiences are expressed. The data are subject to several potential selection biases. Individuals experiencing stronger positive or negative experiences may be more likely to post, while non-participating workers are not represented. In addition, some users may contribute multiple posts, and some content may originate from non-workers (e.g., customers or observers). Differences in platform norms may also contribute to variation across occupational groups. Source, platform, occupation, and period are retained in the analysis to account for heterogeneity across data sources. Country and language information are included only when reliably identifiable. Overall, the findings reflect patterns in sampled online discourse, not population-level estimates of gig-worker sentiment, and should not be interpreted as intrinsic differences between occupational groups.

#### 3.2.5. BERT-Based Text Classification and Topic Identification

The analysis followed a structured text-mining pipeline comprising data preprocessing, sentiment classification, and topic identification. First, the gig-worker texts were pre-processed to remove duplicate records, URLs, irrelevant symbols, and other non-informative content while retaining linguistically meaningful information. The resulting texts were then tokenised using a pretrained BERT tokenizer in accordance with the requirements of the selected model.

For sentiment analysis, a pretrained BERT model was fine-tuned to classify the texts into three sentiment categories: positive, neutral, and negative. The model was trained and evaluated using a predefined training and evaluation procedure, and its classification performance was assessed using accuracy, precision, recall, and F1-score. These metrics were considered jointly to provide a more comprehensive assessment of model performance, particularly given the possibility of class imbalance in the corpus.

In addition to sentiment classification, BERT-based embeddings were generated to represent the semantic characteristics of the texts in a multidimensional vector space. These embeddings were subsequently reduced in dimensionality and subjected to clustering to identify groups of semantically related texts. Topic identification was then based on the resulting clusters. The final number of topics was determined by comparing alternative clustering solutions according to their coherence, interpretability, and stability. This procedure was used to identify thematic structures that were substantively meaningful and sufficiently distinct within the gig-worker corpus.

Overall, the analytical pipeline combines transformer-based sentiment classification with unsupervised semantic clustering to examine both the evaluative orientation and thematic structure of the dataset. This approach provides a systematic framework for analysing large volumes of gig-worker narratives while reducing reliance on manual classification alone.

Nevertheless, automated sentiment classification has inherent limitations. Although BERT-based models can capture contextual information more effectively than many traditional lexicon-based approaches, social media and online forum language remains challenging to classify because of sarcasm, irony, humour, slang, abbreviations, negation, implicit meanings, and context-dependent expressions. Such features may result in classification errors, particularly when the sentiment expressed in a post cannot be reliably determined from the text alone.

To assess these limitations, a subset of posts was manually reviewed, with particular attention to cases involving ambiguous language, sarcasm, irony, humour, and informal expressions. Observed classification errors were categorised according to their predominant error type and considered alongside the overall model-performance metrics. Posts requiring broader conversational or contextual information for interpretation were treated cautiously in the qualitative analysis and were not assumed to provide unambiguous evidence of a particular emotional state.

Accordingly, the BERT predictions are interpreted as probabilistic indicators of sentiment expressed in the text rather than as direct or definitive measures of workers’ underlying emotions or psychological states. This distinction is important because computational sentiment analysis identifies patterns in linguistic expression, whereas the underlying lived experience, emotional state, or psychological condition of an individual cannot be established solely from an online textual post. The findings are therefore interpreted as patterns of expressed sentiment and emotion-related language within the observed corpus rather than as direct measurements of individual workers’ emotions.

#### 3.2.6. Ethical Considerations, Online Data Access, and Data Governance

This study draws on publicly available online texts related to gig work. Importantly, we did not interact with users, participate in online communities, or recruit any individuals. All data were collected passively from open-access sources, and no private, restricted, or password-protected content was accessed or included in the analysis.

From the outset, we designed the study with privacy in mind. We deliberately removed or did not store any direct identifiers such as usernames, profile names, email addresses, or links that could lead back to individual accounts. Instead of focusing on individuals, the analysis works at an aggregate level, meaning that the goal is to understand broader patterns in language and sentiment rather than to profile or trace specific users. No quotes or excerpts are presented in a way that could reasonably allow re-identification.

Ethical approval was not required as the study used only publicly available, non-interactive, and de-identified textual data. All data were handled using strict anonymisation and data minimisation practices throughout the research process.

All data were collected and processed in accordance with the access conditions and terms of use of the respective platforms. We did not attempt to bypass any access restrictions or retrieve non-public information. Likewise, no effort was made to identify individual users or reconstruct personal identities.

Finally, for ethical and legal reasons, the raw dataset is not publicly shared, as redistribution could conflict with platform policies and privacy expectations. However, we provide detailed methodological descriptions, preprocessing steps, and computational procedures in the manuscript to ensure that the study remains transparent and reproducible while still protecting the privacy of individuals whose publicly available texts were analysed.

#### 3.2.7. External Policy Event Registry & Identification Strategy

To eliminate potential endogeneity and circular logic arising from deriving both algorithmic changes and sentiment scores from user forum posts, we constructed an exogenous Platform Policy Event Registry (*N* = 9 major events across 2025). Event dates ($T_{0}$), operational scopes, and policy parameters were identified strictly through external, independently audited sources, including platform newsrooms, corporate filings, and regional regulatory disclosures. Sentiment metrics were subsequently modeled around these externally established cutoff points ($T_{0}$) using Interrupted Time-Series (ITS) and event-study specifications.

## 4. Findings

### 4.1. PRISMA Review Findings

#### 4.1.1. Keyword Co-Occurrence Analysis

To ensure the robustness and validity of the keyword analysis, we further examined whether the results were influenced by the search terms used to construct the dataset. Given that certain core concepts (e.g., “emotion” and “sentiment”) were explicitly included in the search strategy, an additional sensitivity analysis was conducted in which these terms were excluded in order to assess whether the overall thematic structure of the literature would remain stable.

Prior to the analysis, a systematic keyword standardisation procedure was implemented. This involved harmonising spelling variations, unifying singular and plural forms, and consolidating closely related lexical variants (e.g., “emotion” and “emotional,” as well as alternative forms of “well-being”). This preprocessing step ensured conceptual consistency across the dataset and reduced noise arising from superficial linguistic variation.

Subsequently, two separate keyword co-occurrence networks were constructed using identical analytical parameters: (i) a full network including all extracted keywords, and (ii) a restricted network excluding the search-related terms and their directly associated variants. This comparative design enabled an assessment of the extent to which the observed thematic structure was driven by the search strategy versus emerging independently from the underlying literature.

The comparison of the two networks provides a more conservative and methodologically transparent interpretation of the results by distinguishing between themes that are structurally embedded in the literature and those that may be partially induced by the construction of the search query.

The keyword co-occurrence analysis reveals several dominant thematic clusters in the literature. The most central concepts revolve around sentiment analysis, emotion detection, and affective computing, indicating a strong methodological focus on natural language processing techniques. A second cluster captures psychological outcomes such as satisfaction, well-being, and trust, reflecting the behavioral implications of emotional analysis. Finally, emerging themes such as risk perception, stress, and online discourse indicate growing interest in negative emotional dynamics within digital environments.

According to Table 5, the keyword co-occurrence results show that emotion and sentiment form the core of the literature, with the strongest relationship observed between these two terms. This indicates that most studies focus on understanding emotional expressions through sentiment-oriented approaches. Related terms such as emotional also connect to this core, reinforcing the central role of affective dimensions.

Around this core, a second set of relationships links emotions to psychological and evaluative outcomes, including satisfaction, trust, and well-being. Although these connections are less frequent, they suggest that emotions are often studied in relation to how individuals evaluate their experiences. At the same time, the presence of terms such as precarity, insecurity, risk, anger, and fear points to a growing emphasis on negative emotional states. These terms are connected both to emotion and sentiment, indicating that the literature increasingly addresses stress, uncertainty, and vulnerability—especially in digitally mediated or platform-based contexts. Finally, weaker but meaningful links between autonomy and well-being suggest that positive aspects of experience, such as independence and flexibility, are also part of the discussion, although they appear less central compared to emotional and risk-related themes.

According to Table 6, the co-occurrence analysis reveals that sentiment and emotion are the most strongly connected concepts, with a co-occurrence frequency of 358, clearly establishing them as the central focus of the literature. This indicates that most studies are primarily concerned with understanding emotional content through sentiment-oriented approaches.

Table 5 and Table 6 should not be interpreted as reporting the same co-occurrence calculation. They summarize results from two complementary keyword-network analyses conducted as part of the sensitivity assessment. Table 5 reports the pairwise co-occurrence results from the restricted analytical configuration, whereas Table 6 reports the strongest relationships identified in the corresponding full keyword network. Consequently, the co-occurrence frequencies in the two tables are not directly comparable as alternative estimates of the same quantity. The purpose of presenting both tables is to assess the stability of the thematic structure under alternative keyword-processing specifications.

Secondary but still prominent relationships, such as emotion–emotional (99), emotion–affect (51), and sentiment–affect (37), reinforce the centrality of affective dimensions in the research. These connections highlight the field’s strong methodological focus on capturing nuanced emotional experiences, often through natural language processing or computational sentiment analysis techniques.

Another cluster of relationships links emotions to psychological outcomes, including emotion–stress (25), emotion–satisfaction (19), satisfaction–well-being (17), and emotion–well-being (10). These connections suggest that the literature increasingly explores not just the detection of emotions but also their impact on individuals’ subjective experiences and behavioral outcomes.

Finally, the presence of co-occurrences such as emotion–fear (12), emotion–anger (10), and emotional–stress (12) indicates a growing scholarly attention to negative emotional dynamics. These terms suggest that alongside positive outcomes like satisfaction and well-being, the literature is attentive to adverse emotional states, including stress, anxiety, and other affective responses, particularly in digital or platform-mediated environments.

#### 4.1.2. Term Extraction and Frequency Analysis

NLP-based term extraction refers to an automated text-mining approach designed to identify salient terms, multi-word expressions, and domain-specific concepts within a large textual corpus. By relying on computational methods rather than manual coding, this procedure enables a more systematic and efficient identification of relevant linguistic units across extensive datasets. The extracted terms can then be utilised for various analytical purposes, including content analysis, thematic exploration, indexing, and summarisation. In this study, term extraction was employed to capture the most prominent concepts related to algorithmic management and gig workers’ experiences within the analysed corpus.

As presented in Table 7, the existing literature on the algorithmic management of emotions among gig workers is organized into four overarching thematic domains. These domains were identified through the clustering of frequently co-occurring keywords in the reviewed studies, reflecting the underlying intellectual structure of the field. The presence of four distinct clusters suggests that research has evolved around several interconnected yet conceptually differentiated streams. Each cluster represents a coherent area of inquiry characterized by shared theoretical perspectives, methodological approaches, and research interests.

The literature on sentiment analysis is primarily structured around four interconnected research streams. The first stream focuses on the methodological foundations of sentiment and emotion detection using natural language processing and machine learning techniques. The second stream examines psychological and behavioral outcomes, particularly user satisfaction and well-being. A third line of research investigates negative emotional responses such as stress, fear, and anger, often in the context of crisis communication and online discourse. Finally, a growing body of literature explores the role of trust and emotional responses in shaping digital interactions and consumer behavior. Together, these clusters illustrate the multidimensional nature of sentiment analysis research.

### 4.2. Findings of Sentiment Analysis

Sentiment analysis is a critical methodological tool for bridging qualitative and quantitative analysis, enabling robust, data-driven insights into social, behavioural and organisational phenomena. Sentiment analysis has become a pivotal analytical approach for understanding subjective human experiences in environments mediated by digital technologies. In research contexts, sentiment analysis is a powerful way to examine how individuals express perceptions, emotions and evaluations over time and across platforms. Sentiment fluctuated significantly over 12 months.

**Figure 3:**
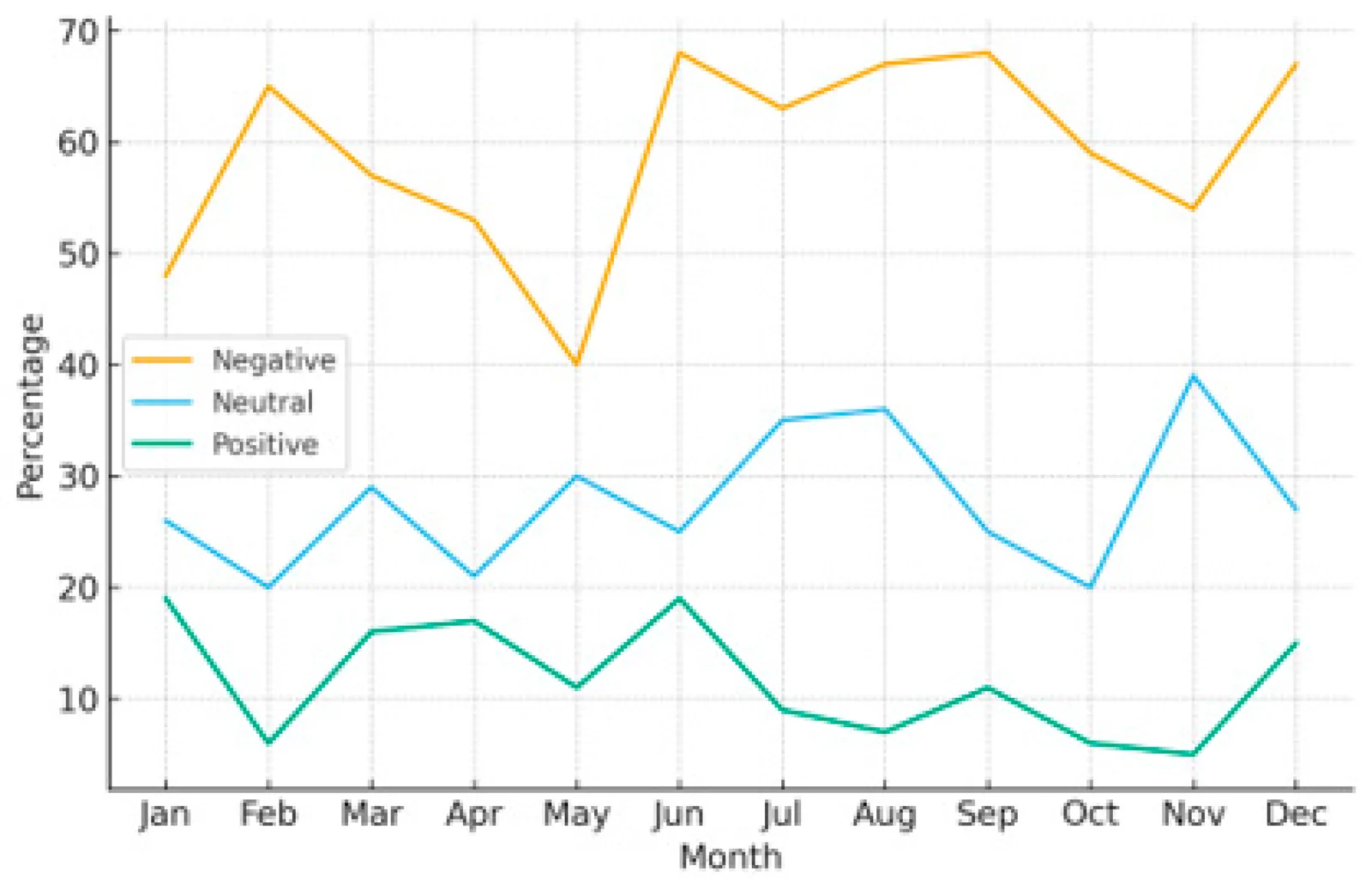
Sentiment Trends Over 12 Months.

According to Figure 3, the sentiment distribution across the 10,000 analyzed posts is dominated by negative sentiment (58%), with neutral (27%) and positive (15%) sentiments occurring less frequently, suggesting a markedly negative overall emotional tone and highlighting potential dissatisfaction or adverse experiences reflected in the data. Negative emotions dominate discussions of algorithmic changes.

Figure 3 presents the monthly distribution of positive, neutral, and negative sentiment across the 12-month observation period. The proportion of negative-affect texts varied across months, ranging from 47.31% in April to 51.49% in September. Across the full 10,000-post corpus, Table 4 reports 4961 negative-affect texts, corresponding to 49.61% of the analytical corpus. Thus, the overall negative-affect proportion was 49.61%, while Figure 3 illustrates month-to-month variation rather than an overall percentage.

**Figure 4:**
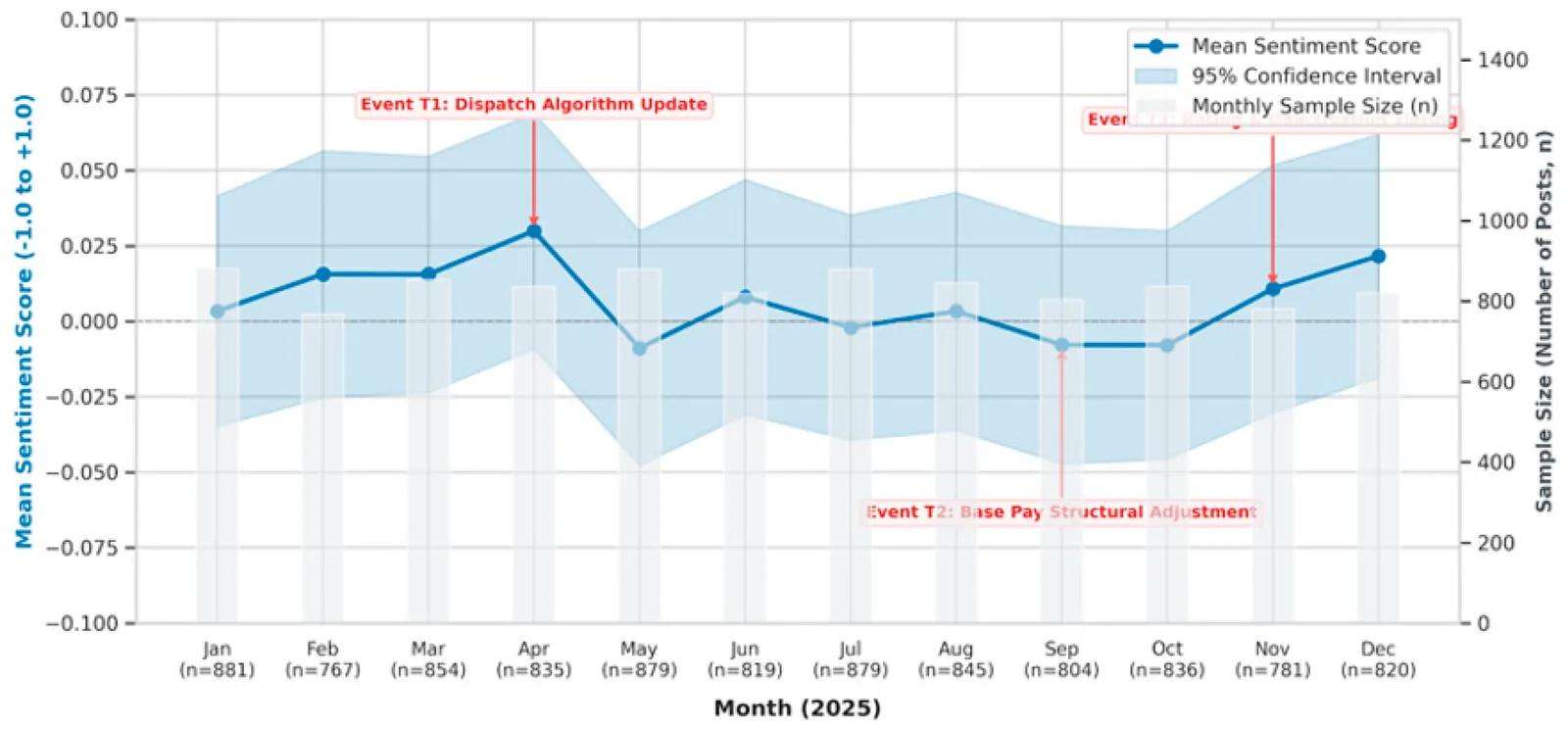
Longitudinal Sentiment Trajectory with Independent Algorithmic Events.

According to Figure 4, longitudinal sentiment trajectory across 10,000 gig worker forum posts (2025). Blue markers indicate monthly mean sentiment scores with shaded 95% confidence intervals ($\bar{x}\pm 1.96\cdot SE$). Grey background bars represent monthly sample sizes (n). Red arrows denote independently verified exogenous algorithmic policy updates ($T_{1},T_{2},T_{3}$), decoupling event identification from textual sentiment measurement.

Negative sentiment peaks were closely associated with specific platform-related events. First, reductions in pay structures triggered strong expressions of anger and perceived betrayal among workers, reflecting heightened sensitivity to income instability. Second, the introduction of new rating systems generated considerable anxiety, with many posts emphasizing concerns over unfair or opaque evaluation mechanisms. Third, automated deactivation waves led to pronounced spikes in fear and confusion, indicating uncertainty regarding job security and the perceived lack of procedural transparency. Finally, changes in algorithmic dispatch systems were frequently interpreted as favoring newly onboarded drivers, fostering perceptions of inequity and systemic bias. Collectively, these findings suggest that key dimensions of algorithmic management—particularly compensation, evaluation, and task allocation—serve as critical triggers of negative emotional responses, reinforcing concerns related to fairness, trust, and worker well-being.

Neutral sentiment dominated during stable algorithmic phases, where no major updates occurred. Positive sentiment spikes were primarily associated with short-term economic incentives and system efficiency improvements. Specifically, temporary bonus promotions and holiday surge pricing generated brief increases in positive expressions, reflecting workers’ responsiveness to immediate financial gains. Additionally, algorithmic enhancements in routing efficiency were linked to improved user experiences, contributing to short-lived satisfaction among workers. However, these positive sentiment periods were not sustained over time, indicating that while financial incentives and operational improvements can temporarily enhance worker sentiment, they do not offset the persistent structural concerns related to compensation, evaluation, and job security. This pattern underscores the transient nature of positive effects in platform-based work environments and highlights the dominance of underlying systemic issues in shaping overall sentiment.

#### 4.2.1. Emotion Category Breakdown

To complement the overall sentiment classification, a fine-grained emotion analysis was conducted to identify the dominant affective states underlying user-generated content. The results indicate that negative sentiment is primarily driven by discrete emotions such as anger, fear, and frustration, while positive sentiment is associated with satisfaction, relief, and hope.

As shown in Table 8, a detailed examination of discrete emotions reveals that workers’ experiences on the platform are dominated by negative affective states. Frustration was the most prevalent emotion, comprising 34% of posts, and was primarily triggered by pay cuts, low order volumes, and technical glitches. Anxiety accounted for 21% of emotional expressions, often arising from fears of deactivation or drops in performance ratings. Anger was observed in 18% of posts, largely in response to sudden algorithmic changes and perceived inequities in order assignments.

Table 8 presents the fine-grained emotion analysis conducted after the broad sentiment classification. Whereas the sentiment analysis distinguishes between positive, neutral, and negative textual orientation, the emotion analysis identifies more specific emotion-related expressions, including frustration, anxiety, anger, hope, satisfaction, and resignation. Accordingly, Table 8 complements the broader sentiment results by showing the specific emotion categories expressed within the corpus.

#### 4.2.2. Relationship Between Algorithmic Updates and Sentiment

Pay Algorithm Updates: Pay reductions elicited the strongest negative emotional reactions among workers, highlighting the central role of compensation in shaping sentiment. Key responses included disillusionment, anger toward perceived “platform greed”, and a notable decline in trust toward the managing platform. Many workers explicitly reported feeling “tricked” or misled by opaque pay formulas, emphasizing the perceived lack of transparency and fairness in compensation structures. These reactions align with broader findings that violations of distributive justice and unclear remuneration mechanisms are potent drivers of sustained negative sentiment in algorithmically managed work environments (Greenberg, 1987; Colquitt et al., 2001).

Ratings and Deactivation Algorithms: Changes to rating systems were a prominent driver of anxiety, reflecting workers’ concerns about the broader implications of algorithmic evaluation. Key emotional responses included fear of customer retaliation, worry about algorithmic misclassification, and heightened concerns regarding job security. These findings suggest that modifications to performance metrics not only influence immediate task outcomes but also trigger sustained psychological stress. Similarly, automated deactivation events were associated with long-lasting negative emotional trajectories, reinforcing workers’ sense of vulnerability and uncertainty. The combination of rating adjustments and deactivation risk underscores the structural pressures inherent in algorithmically managed platforms, where perceived lack of procedural transparency and control amplifies persistent negative sentiment (Hobfoll, 1989; Rosenblat & Stark, 2016).

Routing and Dispatch Algorithms: Gig workers frequently experienced frustration in response to operational inefficiencies and perceived inequities within the platform. Frustration was particularly pronounced when long trips reduced profitability, batch orders increased workload without proportional pay, or priority assignments appeared random or biased. These experiences highlight the central role of perceived fairness in shaping emotional responses, with violations of procedural and distributive justice directly amplifying negative sentiment. The interplay between workload management, task allocation, and fairness perceptions underscores that algorithmic decisions are not merely operational but carry significant emotional and motivational consequences for workers (Colquitt et al., 2001; Greenberg, 1987).

#### 4.2.3. Aggregate Temporal Sentiment Patterns

Looking at sentiment over time, we identified three broad patterns in online discussions about platform work. These patterns differ across types of work and suggest that the overall tone of online conversations shifts depending on job context, platform conditions, and events linked to algorithmic management. It is important to stress that these are group-level patterns in discourse, not emotional histories of individual workers, since the same users are not tracked over time.

Pattern A: “Positive Sentiment in Earlier Observation Periods → Increasing Negative Sentiment” appeared in food-delivery discussions. In earlier periods, posts more often highlighted positive aspects such as earnings, flexibility, and the perceived opportunities of platform work. Over time, however, negative themes became more visible, especially around falling income, heavier workloads, and changes in platform rules. This should be understood as a shift in what is being talked about in the dataset overall, rather than a gradual emotional decline experienced by the same individuals.

Pattern B: “Cyclical Sentiment Fluctuations” was found in ride-hailing discussions. Here, sentiment moved up and down across time. More positive posts tended to appear during periods of surge pricing, bonuses, or better earning opportunities, while more negative posts clustered around regulatory changes, platform updates, and concerns about fairness in task distribution. These ups and downs reflect how discussion topics respond to changing platform conditions, not repeated emotional swings of the same workers.

Pattern C: “Predominantly Neutral Sentiment with Intermittent Anxiety” characterized micro-task discussions, including platforms such as MTurk. Overall, the tone was mostly neutral, but moments of anxiety appeared from time to time. These were often linked to issues like account suspensions, rejected tasks, uncertainty about task availability, and general platform-related risk. This pattern reflects the overall tone of the discussions rather than a stable emotional state of individual workers.

Taken together, these findings show that sentiment in platform-work discussions changes over time and differs across types of work. Some contexts show a gradual shift toward more negative language, others show regular ups and downs, and others remain mostly neutral with occasional spikes of concern. These patterns suggest that platform conditions, economic incentives, and work structures are reflected in how people talk about their experiences online. However, because this study analyzes posts rather than tracking individuals over time, it cannot speak to personal emotional development, long-term dissatisfaction, or individual adaptation. Future research using longitudinal or qualitative approaches would be needed to understand whether these broader discourse patterns also reflect changes in individual workers’ experiences.

## 5. Discussion

### 5.1. Discussion of PRISMA Review Findings

The systematic review provides the theoretical and empirical background for interpreting algorithmic management in platform work. Across the reviewed literature, recurring themes include autonomy and control, well-being and precarity, fairness and trust, algorithmic opacity, performance evaluation, and workers’ responses to platform governance. These findings provide the theoretical context against which the computational analysis of online discourse is interpreted. Importantly, the systematic review and the computational analysis represent different forms of evidence. The review synthesizes findings from prior empirical and theoretical studies, whereas the computational analysis examines patterns of sentiment and emotion-related language in online posts. The two evidence streams are therefore complementary rather than interchangeable.

#### 5.1.1. Well-Being and Worker Experience

Recent research has increasingly examined how algorithmic management influences autonomy, work conditions, and worker experiences in platform-based labor. Platform work is often presented as providing flexibility because workers can decide when and, in some cases, where to work (Rosenblat & Stark, 2016). However, the literature suggests that formal flexibility does not necessarily correspond to substantive autonomy. Task allocation, dynamic pricing, performance ratings, incentive systems, and other algorithmic mechanisms can shape workers’ decisions even when workers formally retain the ability to accept or reject tasks (Lee et al., 2015; Kellogg et al., 2020; Schor et al., 2020).

This tension can be understood as a distinction between formal autonomy and experienced autonomy. Algorithmic systems may simultaneously provide flexibility and introduce constraints. For example, dynamic incentives may allow workers to choose when to work while also encouraging participation during periods of high demand. Similarly, automated task allocation may reduce search costs while limiting workers’ influence over which tasks become available. Algorithmic management should therefore not be treated as uniformly restrictive or enabling; its implications depend on the design, transparency, and consequences of particular algorithmic functions.

The literature also identifies concerns regarding worker well-being, particularly where algorithmic systems involve intensive monitoring, opaque evaluation, unstable earnings, or uncertain access to work (Levy, 2015; Lee, 2018; Wood et al., 2019). These conditions have been associated in prior studies with stress, insecurity, reduced perceived control, and dissatisfaction. At the same time, research also identifies potential benefits associated with flexibility, scheduling autonomy, information access, and coordination (Huws et al., 2017; Lee et al., 2015). The evidence therefore supports a contingent interpretation in which the consequences of algorithmic management depend on how particular systems are designed and experienced.

A related concern is platform precarity. Workers may face fluctuating demand, variable earnings, performance-based access to work, and deactivation risks, while having relatively limited access to traditional employment protections (Wood et al., 2019; Vallas & Schor, 2020; Aloisi & De Stefano, 2022). Algorithmic management can intensify these conditions when important decisions concerning pay, task allocation, or continued platform access are difficult to understand or contest.

These findings are relevant to the present computational analysis because concerns about earnings, ratings, task allocation, uncertainty, and platform rules constitute recurring topics in online discussions. However, the computational analysis does not independently measure worker well-being. References to stress, anxiety, insecurity, or dissatisfaction in online posts are therefore treated as emotion-related or evaluative textual expressions, rather than as validated indicators of psychological outcomes.

#### 5.1.2. Fairness, Organizational Justice, and Trust

Fairness and trust constitute another major theme in the literature on algorithmic management. Algorithmic systems increasingly perform functions traditionally associated with human management, including task allocation, performance evaluation, compensation, ranking, and disciplinary decisions. Their perceived legitimacy therefore depends partly on whether workers consider these processes understandable, consistent, and fair (Lee, 2018; Kellogg et al., 2020).

Organizational justice theory provides a useful framework for interpreting these concerns. Workers may evaluate the fairness of outcomes, such as earnings or task allocation, as well as the fairness of the procedures through which those outcomes are generated (Colquitt et al., 2001). Algorithmic opacity may become particularly consequential when workers cannot understand why a decision occurred or cannot effectively challenge it.

Transparency and explainability may consequently contribute to trust by increasing predictability and reducing uncertainty. Conversely, inconsistent outcomes, unexplained changes, or limited appeal mechanisms may generate distrust and encourage workers to question the legitimacy of platform decisions (Rosenblat & Stark, 2016; Bucher, 2017).

Rating and reputation systems introduce an additional dimension. Although algorithmic systems are frequently presented as objective and data-driven, the data entering these systems may reflect subjective evaluations and social biases. Customer ratings, for example, may contain biases that subsequently influence workers’ access to work or income. Algorithmic processing does not necessarily eliminate such biases and may instead reproduce them through automated decision structures.

The present computational findings are consistent with the discursive salience of these issues. References to pay, ratings, task allocation, platform decisions, and perceived unfairness appear as important themes in the corpus. Nevertheless, the analysis does not constitute a direct measure of individual-level justice perceptions or trust. It demonstrates that fairness-related concerns are visible and repeatedly articulated in online discourse, not that algorithmic management necessarily produces unfairness or distrust among workers as a population.

#### 5.1.3. Negative and Positive Emotion-Related Expressions

The literature identifies a range of affective responses associated with algorithmically mediated work, including frustration, anger, anxiety, fear, satisfaction, and trust. These responses have been discussed in relation to perceived surveillance, uncertainty, reduced autonomy, evaluation, income instability, and limited opportunities for contestation (Lee et al., 2015; Kellogg et al., 2020).

In particular, negative emotion-related expressions may become salient when workers discuss abrupt changes in compensation, task allocation, ratings, or account status. Such situations may be interpreted through organizational justice and COR perspectives as concerns about fairness, predictability, and potential resource loss. However, these theoretical mechanisms should not be interpreted as directly demonstrated psychological processes in the present study.

The distinction between emotion-related language and emotional experience is particularly important. The literature reviewed here includes studies based on surveys, interviews, ethnographic research, and other forms of direct evidence, whereas the computational analysis examines language appearing in online posts. A post containing anxiety-related language may represent a worker’s immediate experience, a hypothetical scenario, advice to another user, exaggeration, humour, or a collective discussion of a broader concern. Consequently, textual classification cannot establish the intensity, persistence, or clinical significance of an emotional state.

Positive expressions also require attention. The literature indicates that algorithmic systems can generate favorable experiences when they improve flexibility, coordination, information access, or earnings opportunities. In the computational corpus, positive expressions associated with bonuses, incentives, favorable demand conditions, or effective platform operations demonstrate that online discourse is not exclusively negative. Such expressions should be interpreted as evidence that positive evaluations are present in the discourse, rather than as evidence of sustained increases in worker satisfaction or well-being.

#### 5.1.4. Risk, Uncertainty, and Autonomy

Risk and autonomy represent closely related dimensions of algorithmically mediated work. Platform workers may face economic, reputational, and algorithmic risks arising from variable earnings, customer ratings, automated evaluations, and uncertain access to future tasks (Aloisi, 2016; Rosenblat & Stark, 2016).

From a COR perspective, income, time, autonomy, and continued access to work can be understood as valued resources. Algorithmic unpredictability may therefore become salient when workers perceive that these resources are difficult to protect or anticipate. Similarly, SDT highlights autonomy and competence as important dimensions through which workers may interpret algorithmically mediated work (Deci & Ryan, 2000; Ryan & Deci, 2017).

The literature nevertheless indicates that autonomy should not be understood as a binary condition. Workers may simultaneously experience greater scheduling flexibility and greater algorithmic dependence. Similarly, algorithmic feedback may help workers understand performance expectations while also increasing the intensity of monitoring. These countervailing possibilities support the contingent theoretical framework adopted in this study.

The computational analysis captures textual references to these conditions but does not directly measure perceived autonomy, resource loss, or risk. Accordingly, references to uncertainty or loss of control are interpreted as discursive representations of these concerns, not as direct measurements of workers’ psychological or economic conditions.

#### 5.1.5. Algorithmic Management and Affective Governance

The concept of affective governance is used in this study to complement, rather than replace, established concepts such as algorithmic control, organizational justice, emotional labor, and algorithmic transparency. Its purpose is to draw attention to how algorithmically mediated working conditions become recurring objects of affective interpretation and public articulation.

Algorithmic systems can generate conditions involving uncertainty, evaluation, reward, visibility, and sanction. These conditions may become affectively salient in workers’ online discussions, particularly when algorithmic decisions have visible consequences for earnings, task access, or platform participation. Affective governance therefore refers here to the discursive and interpretive dimension of algorithmic governance, rather than to direct regulation of workers’ internal emotions.

This distinction is important. The present study does not demonstrate that platforms directly manipulate or control workers’ emotions. Rather, it identifies recurring emotion-related language associated with topics through which algorithmic management is discussed. The observable implication of affective governance in this study is therefore the salience, recurrence, and distribution of affect-related textual expressions.

This formulation also establishes a boundary between affective governance and neighboring concepts. Algorithmic control concerns how computational systems structure and monitor work; organizational justice concerns how workers evaluate fairness; emotional labor concerns the management and display of emotions in work contexts; and affective governance, as used here, focuses on how these algorithmically mediated conditions become affectively interpreted and publicly articulated in digital discourse.

#### 5.1.6. Emotional Consequences of Algorithmic Opacity

Opacity is a recurring issue in the literature on algorithmic management. Workers may have limited knowledge of how task allocation, pricing, evaluation, or deactivation decisions are determined. This can increase uncertainty and make it difficult to anticipate or contest outcomes (Rosenblat & Stark, 2016; Bucher, 2017).

The computational findings indicate that opacity-related concerns are frequently accompanied by frustration, uncertainty, distrust, or other negative emotion-related expressions. This convergence with the literature suggests that algorithmic opacity is a salient topic within online platform-work discourse.

However, the evidence does not demonstrate that opacity causes emotional fatigue, chronic stress, or long-term psychological harm. The more defensible interpretation is that opaque algorithmic practices are repeatedly discussed in affectively charged terms. Whether such discourse corresponds to measurable changes in worker well-being requires direct longitudinal research using validated instruments, interviews, or experience-sampling methods.

#### 5.1.7. Emotional Labor and the Reduced Role of Human Managers

The literature also suggests that algorithmic management changes the social context in which workers receive feedback, evaluation, and support. Automated notifications, ratings, dashboards, and performance indicators increasingly mediate interactions that were traditionally conducted through human supervisors.

In service-oriented platform work, customer ratings and automated evaluations may also influence how workers manage their interactions and emotional displays. Emotional labor therefore remains relevant to platform work, although the mechanisms differ from those found in conventional organizational settings.

The present study does not directly measure emotional labor. Instead, the literature review identifies it as an important theoretical mechanism, while the computational analysis captures textual expressions that may refer to experiences of evaluation, pressure, frustration, or interaction with customers and platforms. The absence of direct observation means that claims concerning emotional regulation should remain theoretical rather than empirical conclusions of this study.

### 5.2. Discussion of Computational Sentiment Analysis Findings

The computational analysis provides a complementary perspective on the systematic review by examining how platform work is represented and evaluated in online discourse. The central finding is that negative sentiment and negative emotion-related language are prominent in the corpus, particularly around issues involving compensation, algorithmic evaluation, task allocation, uncertainty, and perceived unfairness. However, these findings should be interpreted as properties of online discourse rather than direct measurements of workers’ emotional states or well-being.

The prominence of negative language may partly reflect the communicative characteristics of online communities. Individuals experiencing problems may have stronger incentives to seek advice, complain, or warn others, while routine or positive experiences may be less likely to generate posts. Negativity bias may contribute to the greater salience and processing of adverse experiences relative to positive experiences, potentially increasing their prominence in online discourse (Baumeister et al., 2001). The observed negative sentiment therefore provides information about what is salient and discussable within the sampled communities, rather than an estimate of how workers generally feel.

#### 5.2.1. Sentiment, Fairness, and Algorithmic Evaluation

A substantial proportion of the negative discourse concerns pay, ratings, task allocation, account decisions, and platform rules. These themes correspond closely with constructs identified in the systematic review, particularly organizational justice, resource security, and algorithmic uncertainty.

From a justice perspective, discussions concerning compensation and task allocation may reflect attempts by contributors to evaluate whether platform outcomes are fair. From a COR perspective, references to income instability or deactivation may represent concerns about valued resources. Nevertheless, the computational analysis cannot determine whether a particular contributor experienced an objectively unfair outcome or suffered an actual resource loss.

The appropriate conclusion is therefore that fairness- and resource-related concerns are prominent in the textual discourse, not that algorithmic management objectively causes injustice or psychological insecurity.

#### 5.2.2. Emotion-Related Language and Interpretive Boundaries

The emotion classification identifies expressions associated with frustration, anger, anxiety, hope, satisfaction, and resignation. These categories provide a more detailed description of the affective vocabulary present in the corpus than broad positive-neutral-negative sentiment scores alone.

Nevertheless, an emotion label attached to a post should not be interpreted as a validated measure of the author’s internal emotional state. Online communication can include sarcasm, humour, exaggeration, quotation, hypothetical statements, advice, collective narratives, and references to other people’s experiences. Emotion-related textual expressions are therefore best understood as linguistic indicators of affective discourse.

This distinction is central to the contribution of the study. The analysis identifies what kinds of emotional language appear in discussions of platform work and how that language varies across sources, occupations, and periods. It does not estimate the prevalence or severity of emotional states among the broader population of gig workers.

#### 5.2.3. Positive Sentiment and Countervailing Evidence

The presence of positive sentiment is theoretically important because it prevents the computational findings from being interpreted as evidence that algorithmic management is inherently harmful. Positive expressions occur in discussions concerning favorable earnings, bonuses, flexible schedules, efficient task allocation, and temporary improvements in platform conditions.

These findings are consistent with the beneficial pathway specified in the a priori framework. Transparent information, predictable rewards, efficient matching, and flexible scheduling may generate favorable evaluations of algorithmically mediated work.

At the same time, the computational analysis cannot establish whether such positive expressions represent sustained satisfaction or improved well-being. Positive sentiment may reflect a particular event, temporary financial gain, or favorable comparison with previous conditions. The findings therefore support a contingent and non-deterministic interpretation rather than a simple positive-negative dichotomy.

#### 5.2.4. Temporal Variation Without Individual Trajectories

Temporal analysis indicates that sentiment varies across the 12-month observation period. In some occupationally associated discussion spaces, negative expressions become more prominent during particular periods, while positive or neutral expressions increase during others.

These patterns may correspond to platform-related events, policy discussions, incentive periods, seasonal changes, regulatory developments, or collective attention cycles. However, because the data do not constitute an individual-level panel, the analysis cannot determine whether the same worker experienced successive emotional stages.

Consequently, the study does not claim to demonstrate long-term emotional trajectories, chronic dissatisfaction, emotional decline, or individual adaptation. The appropriate unit of interpretation is the temporal distribution of online discourse. The findings indicate that the content and tone of public discussion change over time, not that individual workers necessarily undergo corresponding psychological changes.

#### 5.2.5. Occupational and Source Differences

Differences across food-delivery, ride-hailing, and micro-task discussions should also be interpreted cautiously. Occupational environments differ in task structures, earnings models, customer interaction, algorithmic controls, and platform dependence. However, occupational groups are also represented through different online communities and communication environments.

Accordingly, observed differences may reflect both occupational conditions and source-specific communication practices. Reddit, groups, worker forums, and review platforms may differ in their user populations, moderation rules, norms of complaint, and expectations concerning self-presentation. The findings therefore represent differences in occupationally associated online discourse, rather than definitive evidence that workers in one occupation experience more negative emotions than workers in another.

The source-level and sensitivity analyses provide additional information about the robustness of the observed patterns. Nevertheless, no statistical adjustment can completely eliminate the possibility of selection into online communities or differences in communicative norms.

### 5.3. Integration of the Systematic Review and Computational Evidence

The systematic review and computational analysis make distinct but complementary contributions to the study. The systematic review synthesises theoretical and empirical evidence concerning algorithmic management, including autonomy, fairness, resource insecurity, transparency, evaluation, and worker experiences. In contrast, the computational analysis examines whether related concepts and emotion-related expressions are observable within a large corpus of online gig-worker discourse. Thus, the computational analysis does not seek to validate the findings of the systematic review directly; rather, it provides complementary evidence concerning how these themes are expressed and discussed in online environments. Table 9 integrates the findings of the systematic review and computational analysis by comparing the principal theoretical themes identified in the literature with their manifestation in online gig-worker discourse.

The integration should be understood as complementary rather than confirmatory: the systematic review identifies patterns established in prior research, whereas the computational analysis identifies patterns in expressed online discourse. Therefore, convergence between the two evidence streams does not by itself establish causal relationships or demonstrate that online sentiment directly reflects workers’ lived experiences.

## 6. Implications

The practical implications of this study should be interpreted as theoretically informed governance and design implications rather than interventions empirically validated by the present analysis. The computational findings identify recurring concerns in online discourse, while the systematic review provides broader evidence concerning the conditions under which algorithmic management may be experienced as enabling or constraining.

### 6.1. Implications for Platform Design and Governance

The findings support greater attention to transparency and predictability in consequential algorithmic decisions. Platforms could provide advance communication concerning substantial changes to compensation, task allocation, evaluation criteria, and other rules that materially affect workers’ access to income or work.

Clear explanations of automated decisions are also important. Where algorithmic systems determine ratings, task allocation, compensation, or account status, workers should be provided with understandable information concerning the basis of consequential decisions.

Meaningful human review and appeal mechanisms represent a further priority. Automated decisions that materially affect continued access to platform work should be contestable through accessible procedures involving appropriate human oversight.

Independent auditing may provide an additional governance mechanism. Rating, dispatch, pricing, evaluation, and deactivation systems could be periodically assessed for consistency, unintended disparities, explainability, and potentially arbitrary outcomes.

Platforms should also provide appropriate safeguards against arbitrary suspension or deactivation. Clear criteria, notification, explanation, and meaningful opportunities for appeal may strengthen procedural legitimacy without assuming that these measures will automatically improve psychological well-being.

### 6.2. Implications for Policymakers and Institutions

For policymakers, the findings highlight the importance of governance mechanisms that address transparency, accountability, and contestability in algorithmically mediated labor markets. Regulatory approaches may consider requirements for advance notification of consequential algorithmic changes, explanations of automated decisions, access to human review, and protection against arbitrary deactivation.

Worker participation should also be considered. Workers possess practical knowledge of how algorithmic systems operate in everyday contexts and may identify unintended consequences that are difficult to anticipate during system design. Mechanisms for worker consultation or collective representation may therefore contribute to more accountable algorithmic governance.

Access to relevant platform-level information is similarly important for meaningful oversight. Where feasible, researchers, regulators, worker representatives, and other legitimate stakeholders should have appropriate access to information needed to evaluate consequential algorithmic systems.

These recommendations should be viewed as governance propositions informed by the literature and the observed discourse, not as interventions demonstrated by this study to reduce negative sentiment or improve worker well-being.

### 6.3. Implications for Gig Workers

The implications for individual workers require particular caution. Strategies such as participating in online communities, monitoring platform rules, or using multiple platforms may sometimes help workers manage uncertainty. However, these strategies can also increase administrative effort, unpaid coordination time, cognitive burden, and exposure to multiple algorithmic systems.

For this reason, the study does not present worker adaptation as a substitute for platform accountability. Individual coping strategies may help workers navigate existing conditions, but responsibility for transparency, fair procedures, explainability, and protection against arbitrary decisions should remain primarily with platforms and relevant institutional actors.

### 6.4. Overall Implication

Taken together, the findings support a shift from viewing algorithmic management solely as a technical mechanism of coordination toward understanding it as a form of socio-technical governance whose consequences depend on design, context, transparency, and contestability.

The evidence does not establish that algorithmic management inevitably produces negative emotional outcomes. Instead, it indicates that particular algorithmic practices—especially those involving opacity, uncertainty, evaluation, compensation, and access to work—are recurrently discussed in affectively charged terms. The practical challenge is therefore not to eliminate algorithmic management but to design and govern it in ways that increase predictability, transparency, procedural fairness, worker participation, and meaningful recourse.

## 7. Limitations

Despite the insights this study offers, several limitations should be considered when interpreting the findings. The dataset is not a probability sample, as participation in online platforms is voluntary and therefore self-selected. As a result, the corpus may disproportionately reflect more active users or individuals with particularly strong, often negative, experiences, which limits the generalisability of the findings to the broader population of gig workers. Moreover, the sentiment expressed in online posts should not be interpreted as a direct representation of workers’ lived experiences or psychological well-being, but rather as a form of self-presentation shaped by platform-specific norms, communicative intentions, and contextual factors. Although the data cover a 12-month period, the same individuals are not consistently tracked over time; consequently, the longitudinal analysis reflects changes in aggregate discourse patterns rather than within-individual changes in attitudes or emotions.

In addition, variations in posting frequency, combined with the absence of stable user identifiers, may lead to disproportionate influence from highly active contributors, making it difficult to clearly separate individual-level behaviour from broader collective patterns. The use of multiple online platforms further introduces heterogeneity, as each platform operates under distinct norms, moderation practices, and user communities, all of which may shape observed patterns across occupational and temporal dimensions. Furthermore, key contextual variables such as occupation, country, and language cannot always be verified with full accuracy, introducing potential classification uncertainty and unobserved heterogeneity. The focus on selected gig-work sectors also limits the extent to which the findings can be generalised to other forms of platform labour, such as freelance digital work or remote knowledge-based services.

Finally, while automated sentiment analysis enables the identification of large-scale textual patterns, it cannot fully capture the complexity of human language, including sarcasm, irony, ambiguity, and culturally specific meanings; therefore, the results should be interpreted as broad indicators of sentiment rather than precise reflections of workers’ subjective experiences.

## 8. Conclusions

This study examines how algorithmic management in gig work is discussed in online discourse and how these discussions vary across platforms and over time. By combining a PRISMA-based systematic literature review with computational analysis of online discussions, it explores how themes such as platform work, algorithmic control, earnings, fairness, uncertainty, and working conditions are represented in both academic research and everyday online conversations (Kellogg et al., 2020; Rosenblat & Stark, 2016).

For the first research question, the findings indicate that both academic scholarship and online discussions converge on a set of core concerns, including control, transparency, income instability, and fairness. However, these shared themes are framed differently across the two domains. Academic studies predominantly adopt a structural and organisational perspective, focusing on platform design, governance, and institutional arrangements (Kellogg et al., 2020; Vallas & Schor, 2020). By contrast, online discussions are more grounded in lived experience, foregrounding uncertainty, fluctuating earnings, and the practical challenges of gig work (Wood et al., 2019). Thus, while there is thematic overlap, the emphasis differs between analytical academic framings and experience-based narratives.

For the second research question, the analysis reveals systematic differences across platform types. Discussions relating to delivery and ride-hailing work tend to exhibit more negative language than those associated with micro-task platforms. This negativity is particularly associated with issues such as remuneration, workload, and perceived unfairness in platform decision-making. Emotion-related results further indicate that expressions of frustration and dissatisfaction are more prevalent in contexts characterised by stronger algorithmic control and greater income uncertainty. Overall, these patterns suggest that platform structure and the nature of work are reflected in the emotional tone of online discourse (Rosenblat & Stark, 2016; Wood et al., 2019).

For the third research question, the results demonstrate that sentiment is not static but fluctuates over the 12-month observation period. Periods of increased negative sentiment often coincide with events such as platform policy changes, modifications to payment systems, or broader public debates concerning labour conditions within online communities. However, these temporal variations should be interpreted as aggregate patterns in online discourse rather than stable emotional states of individual workers, as the dataset does not track the same individuals longitudinally. This limitation aligns with prior work cautioning against inferring psychological states from large-scale textual data (Golder & Macy, 2011; Hovy & Spruit, 2016).

Overall, the findings indicate that sentiment and emotion-related language varies across both platform types and time periods. Negative expressions are more frequently associated with discussions of earnings, workload, policy changes, and fairness. At the same time, these patterns should be understood as reflections of how gig work is articulated in online environments, rather than as direct evidence of workers’ lived emotional experiences. The analysis does not demonstrate that algorithmic management directly affects workers’ emotions or long-term psychological well-being, nor does it capture individual emotional trajectories over time (Kellogg et al., 2020).

The principal contribution of this study is therefore to map how algorithmic management is represented and emotionally framed in online discourse, rather than to measure workers’ subjective emotional states. By integrating a systematic literature review with computational text analysis, the study provides a broader account of how platform work is constructed, interpreted, and evaluated across both academic and public-facing discussions.

It is also important to emphasise that the sentiment and emotion measures employed in this study capture linguistic patterns in text rather than psychological conditions. Sentiment scores reflect the overall evaluative orientation of language, while emotion categories identify the presence of emotion-related expressions within posts. These measures should not be interpreted as direct indicators of mental health, stress, or clinical emotional states (Calvo & D’Mello, 2010; Mohammad, 2016).

Online discussions are shaped by a range of communicative and contextual factors beyond individual experience. Users may post to seek advice, report incidents, express frustration, share positive experiences, or employ humour and sarcasm. Consequently, the emotional tone of online discourse does not necessarily correspond to the full spectrum of workers’ everyday experiences. In addition, negative experiences may be more likely to be shared publicly, potentially increasing the visibility of negative sentiment relative to neutral or positive expressions (Golder & Macy, 2011).

For these reasons, the results should be interpreted as patterns in how gig work is discussed in online settings, rather than as direct evidence of workers’ emotional well-being. They illustrate how platform work is narrated and evaluated across different contexts and over time, but they do not indicate whether individual workers consistently experience stress, frustration, or satisfaction in their daily working lives.

## 9. Future Research Directions

Future research should extend the present findings through longitudinal, comparative, and mixed-method designs. First, individual-level panel studies are needed to distinguish within-person changes in sentiment, perceived fairness, autonomy, and well-being from aggregate temporal patterns. Experience-sampling and diary methods could further improve temporal precision by capturing workers’ responses closer to algorithmic interventions.

Second, comparative studies across platforms, occupations, and national contexts would help identify whether observed patterns reflect platform-specific dynamics or broader mechanisms of algorithmic management. In addition, mixed-method approaches combining computational analysis with interviews and ethnography could strengthen interpretation by explaining how workers experience and respond to algorithmic governance.

Third, intervention-oriented and quasi-experimental designs are required to assess whether transparency, explainability, human oversight, and participatory governance improve worker outcomes and to better approximate causal relationships where data allow.

Finally, future research should further examine the intersection of algorithmic management, emotional labour, and precarity, as well as employ multilevel longitudinal models to separate individual-, platform-, and context-level effects.

Overall, the present study captures how algorithmically mediated work is collectively discussed and affectively expressed online, while individual-level psychological processes and causal mechanisms require further investigation.

## Figures and Tables

**Table 1:** PRISMA Records by Database.

Databases	Number of Records
Google Scholar	598
IEEE Xplore	634
PubMed	619
SSRN	559
Scopus	602
Web of Science	582
ACM DL	112
Total	3706

**Table 2:** Data Retrieval, Screening, and Final Analytical Corpus.

Data-Generation Stage	Number of Records	Percentage
Raw retrieved records	24,258	100.00%
Records excluded during screening and eligibility assessment	14,258	58.75%
Eligible records	10,000	41.25%
Final analytical corpus	10,000	41.25%

**Table 3:** Monthly Distribution of the Final 10,000-Post Analytical Corpus Across Platforms.

Month	Bolt	DoorDash	Getir	Gojek	Grab	Uber	Yemeksepeti	Total
Jan	137	125	146	127	110	128	108	881
Feb	118	116	94	93	126	107	113	767
Mar	110	132	113	123	122	117	137	854
Apr	101	128	116	126	119	114	131	835
May	126	138	136	129	96	128	126	879
Jun	113	118	135	108	111	113	121	819
Jul	121	113	142	144	116	115	128	879
Aug	117	142	113	114	115	125	119	845
Sep	103	128	124	96	110	116	127	804
Oct	126	111	107	112	124	113	143	836
Nov	86	136	115	95	124	104	121	781
Dec	110	100	117	115	130	113	135	820
Total	1368	1487	1458	1382	1403	1393	1509	10,000

**Table 4:** Monthly Distribution of Gig-Worker Texts and Negative Affect During the Observation Period (January–December 2025).

Month (2025)	Total Texts (N)	Negative Affect Texts (n)	% Negative Affect	Mean Sentiment ($\bar{Y}$)	Std. Error (SE)	95% CI of Mean Sentiment
**Jan**	881	443	50.28%	+0.0034	0.0195	[−0.0348, +0.0416]
**Feb**	767	379	49.41%	+0.0156	0.0208	[−0.0252, +0.0565]
**Mar**	854	412	48.24%	+0.0156	0.0200	[−0.0236, +0.0547]
**Apr**	835	395	47.31%	+0.0299	0.0199	[−0.0091, +0.0690]
**May**	879	441	50.17%	−0.0089	0.0198	[−0.0476, +0.0299]
**Jun**	819	407	49.69%	+0.0080	0.0199	[−0.0309, +0.0470]
**Jul**	879	439	49.94%	−0.0020	0.0190	[−0.0393, +0.0352]
**Aug**	845	424	50.18%	+0.0033	0.0201	[−0.0361, +0.0427]
**Sep**	804	414	51.49%	−0.0078	0.0201	[−0.0472, +0.0315]
**Oct**	836	428	51.20%	−0.0079	0.0193	[−0.0457, +0.0300]
**Nov**	781	381	48.78%	+0.0107	0.0210	[−0.0303, +0.0518]
**Dec**	820	398	48.54%	+0.0216	0.0206	[−0.0187, +0.0619]
**Annual Total/Avg.**	10,000	4961	49.61%	+0.0066	0.0058	[−0.0047, +0.0179]

**Table 5:** Findings of Keyword Co-occurrence.

Term 1	Term 2	Co-Occurrence
emotion	sentiment	151
emotion	emotional	6
autonomy	well-being	3
emotion	support	3
sentiment	support	3
emotional	sentiment	3
precarity	well-being	2
emotion	trust	2
fairness	trust	2
emotion	risk	2
emotion	satisfaction	2
satisfaction	sentiment	2
insecurity	satisfaction	2
anger	fear	2
anger	sentiment	2
fear	sentiment	2
risk	sentiment	2
emotional	support	2
autonomy	wellbeing	1
autonomy	emotional	1

**Table 6:** The Strongest Co-occurrence Relationships.

Term 1	Term 2	Co-Occurrence
sentiment	emotion	358
emotion	emotional	99
emotion	affect	51
sentiment	affect	37
emotion	stress	25
emotion	satisfaction	19
sentiment	emotional	18
satisfaction	well-being	17
emotion	feeling	15
emotional	affect	14
emotion	fear	12
emotional	stress	12
sentiment	satisfaction	12
emotion	anger	10
emotion	well-being	10

**Table 7:** Frequencies of Themes.

Term	Frequency
emotion	404
sentiment	380
affect	69
emotional	58
well-being	34
trust	26
stress	21
satisfaction	20
feeling	11
fear	5
anger	6
anxiety	4
happiness	1

**Table 8:** Emotion Analysis Results.

Emotion Category	Percentage	Example Triggers
Frustration	34%	Pay cuts, low orders, technical glitches
Anxiety	21%	Deactivation risk, rating drops
Anger	18%	Sudden algorithm changes, order assignment issues
Hope	12%	Rumors of new bonuses, promotional periods
Satisfaction	9%	Successful high-earning days
Resignation	6%	Long-term adaptation to poor conditions

**Table 9:** Integration of Systematic Review and Computational Analysis Findings.

Analytical Dimension	Systematic Review Findings	Computational Analysis Findings	Integrated Interpretation
Algorithmic Control	Algorithmic management is commonly associated with automated evaluation, task allocation, dynamic pricing, information asymmetry, and automated sanctions.	Online discourse frequently refers to algorithmic decisions concerning task allocation, performance evaluation, earnings, and sanctions.	Both evidence streams indicate that algorithmic control is a central feature shaping workers’ perception of platform work.
Autonomy	The literature presents platform work as offering flexibility while also identifying forms of constrained or illusory autonomy.	Online discussions contain both positive references to flexibility and negative expressions concerning limited control over tasks, schedules, and platform decisions.	The two evidence streams suggest that perceived autonomy depends on the extent to which algorithmic systems constrain workers’ practical control.
Fairness and Justice	Organizational justice and fairness are frequently identified as important mechanisms linking algorithmic management to worker attitudes and well-being.	Negative online expressions frequently concern perceived unfairness, inconsistent decisions, opaque evaluations, and unequal treatment.	Computational evidence provides discourse-level support for the prominence of fairness-related concerns identified in the literature.
Transparency and Explainability	Prior research emphasizes transparency, procedural fairness, and explainability as important conditions for trust in algorithmic systems.	Online discussions frequently express frustration or uncertainty when workers cannot understand algorithmic decisions or obtain explanations.	Both analyses highlight transparency and explainability as important dimensions of workers’ responses to algorithmic management.
Emotional Responses	The literature identifies frustration, anxiety, dissatisfaction, stress, and, under certain conditions, positive affect and satisfaction.	Negative sentiment predominates in the analysed corpus, with frustration, anxiety, and anger representing prominent expressed emotions.	Computational findings broadly correspond with the literature while indicating that negative affect is particularly visible in online worker discourse.
Well-Being	Algorithmic control has been associated with psychological strain, stress, reduced well-being, and uncertainty, although some studies report beneficial effects under supportive conditions.	Negative sentiment and emotion expressions frequently concern uncertainty, pressure, financial insecurity, and dissatisfaction.	The findings suggest a consistent association between perceived algorithmic constraints and negative affective expressions, while not establishing individual-level effects on psychological well-being.
Precarity and Economic Insecurity	The literature links platform work to income uncertainty, employment insecurity, and limited social protection.	Online discourse includes concerns about fluctuating earnings, unstable demand, incentives, penalties, and dependence on platform decisions.	Both evidence streams identify economic insecurity as an important contextual factor shaping worker experiences of algorithmic management.
Trust and Acceptance	Trust in platforms is associated with perceived fairness, predictability, transparency, and accountability.	Negative expressions frequently emerge around unpredictable decisions, opaque policies, and limited opportunities to contest decisions.	The combined evidence indicates that transparency, predictability, and procedural fairness are important conditions for trust and acceptance.
Temporal Patterns	The literature provides evidence of changing worker perceptions as algorithmic practices evolve, but individual-level longitudinal evidence remains limited.	Monthly analysis identifies changes in the aggregate tone and composition of online discourse over the 12-month observation period.	The computational analysis extends the literature by identifying temporal variation in expressed sentiment, while not establishing within-person emotional trajectories or causal effects.
Worker Heterogeneity	Prior studies suggest that platform type, occupation, worker characteristics, and institutional context can moderate responses to algorithmic management.	Sentiment patterns vary across occupational and source contexts, although source composition and participation differences may also contribute to these variations.	The findings support the importance of contextual heterogeneity while highlighting the need for worker-level and comparative research.
Overall Convergence	The literature predominantly identifies fairness, autonomy, transparency, precarity, and well-being as central dimensions of algorithmic management.	Computational evidence identifies corresponding patterns in online worker discourse, with negative sentiment particularly prominent.	The two evidence streams are broadly convergent, with computational analysis providing complementary discourse-level evidence rather than direct validation of workers’ lived experiences.

Source: Authors’ own elaboration.

## Data Availability

The corresponding author can provide the datasets created and/or analyzed during this investigation upon reasonable request, but they are not publicly accessible.

## Supplementary Materials

The following supporting information can be downloaded at: https://www.mdpi.com/article/10.3390/bs16091709/s1, S1. PRISMA 2020 Checklist, S2. PRISMA Records and Screening Information.
